# An evaluation of how connectopic mapping reveals visual field maps in V1

**DOI:** 10.1038/s41598-022-20322-4

**Published:** 2022-09-28

**Authors:** David M. Watson, Timothy J. Andrews

**Affiliations:** Department of Psychology and York Neuroimaging Centre, University of York, York, YO10 5DD UK

**Keywords:** Striate cortex, Computational neuroscience

## Abstract

**Abstract:**

Functional gradients, in which response properties change gradually across the cortical surface, have been proposed as a key organising principle of the brain. However, the presence of these gradients remains undetermined in many brain regions. Resting-state neuroimaging studies have suggested these gradients can be reconstructed from patterns of functional connectivity. Here we investigate the accuracy of these reconstructions and establish whether it is connectivity or the functional properties within a region that determine these “connectopic maps”. Different manifold learning techniques were used to recover visual field maps while participants were at rest or engaged in natural viewing. We benchmarked these reconstructions against maps measured by traditional visual field mapping. We report an initial exploratory experiment of a publicly available naturalistic imaging dataset, followed by a preregistered replication using larger resting-state and naturalistic imaging datasets from the Human Connectome Project. Connectopic mapping accurately predicted visual field maps in primary visual cortex, with better predictions for eccentricity than polar angle maps. Non-linear manifold learning methods outperformed simpler linear embeddings. We also found more accurate predictions during natural viewing compared to resting-state. Varying the source of the connectivity estimates had minimal impact on the connectopic maps, suggesting the key factor is the functional topography within a brain region. The application of these standardised methods for connectopic mapping will allow the discovery of functional gradients across the brain.

**Protocol registration:**

The stage 1 protocol for this Registered Report was accepted in principle on 19 April 2022. The protocol, as accepted by the journal, can be found at 10.6084/m9.figshare.19771717.

## Introduction

Functional gradients are an important organising principle in the brain^1^. The key feature underlying these gradients is a gradual change in preferred stimulus or response parameters that is topographically mapped across brain regions. Gradients are well described in primary sensory regions, such as retinotopic maps in visual cortex^2–4^, somatotopic maps in somatosensory cortex^5,6^, and tonotopic maps in auditory cortex^7–9^. Functional gradients have also been proposed outside of primary sensory regions, including topographic maps of real-world object size^10^ and animacy^11^ in the ventral visual stream, and maps of numerosity^12^ and multisensory integration^13^ in the dorsal visual stream. Nevertheless, the majority of research to date has regarded regions outside of primary sensory cortices as comprising distinct parcels or modules^14^, and the role of underlying functional gradients has been frequently overlooked (although see^15,16^). Consequently, functional gradients throughout many brain regions are poorly understood, while others yet may remain entirely undiscovered. While there are established paradigms for measuring maps in primary sensory regions, such as visual field mapping in visual cortex^17,18^, it is often unclear what tasks would be required to estimate gradients in other regions.

The function of a brain region is closely tied to its connectivity with other regions, such that functionally similar neurons may also be expected to show similar connectivity patterns^19,20^. Consequently, the topographic organisation of brain functions may be similarly reflected in a topographic organisation of connectivity^21^, yielding so-called “connectopic” maps^22,23^. As such, functionally similar points within a brain region may be represented close to one another within the corresponding connectivity space, while functionally dissimilar points may appear more distant. Thus, each of these points may be distributed along a manifold within the connectivity space, with the principal dimensions of that manifold corresponding to the principal functional gradients underpinning that brain region. This implies that functional gradients may be reconstructed from connectivity patterns by extracting the principal dimensions from within the connectivity space. Particular focus has been given to manifold learning techniques that extract non-linear dimensions^24,25^. By applying manifold learning to resting-state functional connectivity patterns, previous studies have identified coarse-scale gradients spanning the whole brain^16,25^, as well as local gradients within brain regions including the striatum^26^, hippocampus^27^, and primary sensory regions^22,28^. The same techniques can also be applied to recover functional gradients from structural connectivity^29^. Thus, connectopic mapping provides a potential method for characterising functional gradients without necessarily requiring prior knowledge of those gradients or a specific task to elicit them.

Nevertheless, there remain a number of open questions regarding the efficacy and practicalities of connectopic mapping. Firstly, previous studies have not provided ground-truth estimates of the functional gradients to benchmark the connectopic maps against. For instance, while Haak and colleagues^22^ demonstrated that connectopic mapping can reconstruct retinotopic maps in primary visual cortex, they did not have access to maps estimated directly from traditional visual field mapping techniques to compare these reconstructions against. Consequently, it remains unclear how accurately the functional gradients are reconstructed in each individual. Secondly, previous connectopic mapping studies have exclusively measured functional connectivity in the brain at rest. While resting-state estimates are a common choice, they have also been criticised as representing an unnatural cognitive state that may not generalise well to more active states^30^. An alternative approach is to measure functional connectivity during naturalistic stimulation (e.g. movie-watching)^31^, and indeed such paradigms may yield more reliable connectivity patterns in some circumstances^32,33^. Finally, many algorithms exist for extracting the principal dimensions of the connectivity space, yet a comprehensive comparison between them remains lacking. Previous studies have primarily employed spectral embedding^22,26,28,29^ and diffusion map^16,27^ manifold learning techniques, but many other such techniques exist. Manifold learning techniques will likely outperform linear embeddings if the representation is non-linear, but linear techniques are typically simpler and may still perform well if the representation is linear.

Here, we provide a comprehensive assessment of connectopic mapping for reconstructing local functional gradients, using the retinotopic maps in primary visual cortex as a test case. We compare the performance of multiple commonly used dimensionality reduction techniques by benchmarking the reconstructions against retinotopic maps derived from traditional visual field mapping techniques. We give particular focus to spectral embedding as this has previously been applied to reconstructing gradients in sensory regions^22,28^, though we also consider a number of other linear embeddings and manifold learning algorithms. In an initial experiment, we perform exploratory analyses of a publicly available naturalistic-imaging dataset^34^. We then provide a preregistered replication experiment using both resting-state and naturalistic-imaging data obtained from the Human Connectome Project^35^. If connectopic mapping provides a robust reconstruction of the underlying functional gradients, we would expect a high degree of correspondence between the maps estimated from connectivity and those-measured directly by visual field mapping. By developing standardised connectivity methods, it will be possible to determine whether topographic maps are a ubiquitous organising principle across the brain.

## Results

### Experiment 1

We first performed exploratory analyses of 15 subjects obtained from the publicly available StudyForrest naturalistic-imaging MRI dataset^34,36,37^ (https://www.studyforrest.org/). This includes approximately 2 h of movie-watching data plus retinotopic mapping scans for each subject. We first extracted visual field maps in each subject via a travelling-wave analysis^17^ of the retinotopy data. These were used to define individualised V1 regions of interest (ROIs) on the cortical surface, identified by tracing along the phase reversals in the polar angle map. The eccentricity and polar angle phase maps within V1 also served as ground-truth estimates of the visual field maps, which the connectopic maps could be compared against.

**Figure 1:**
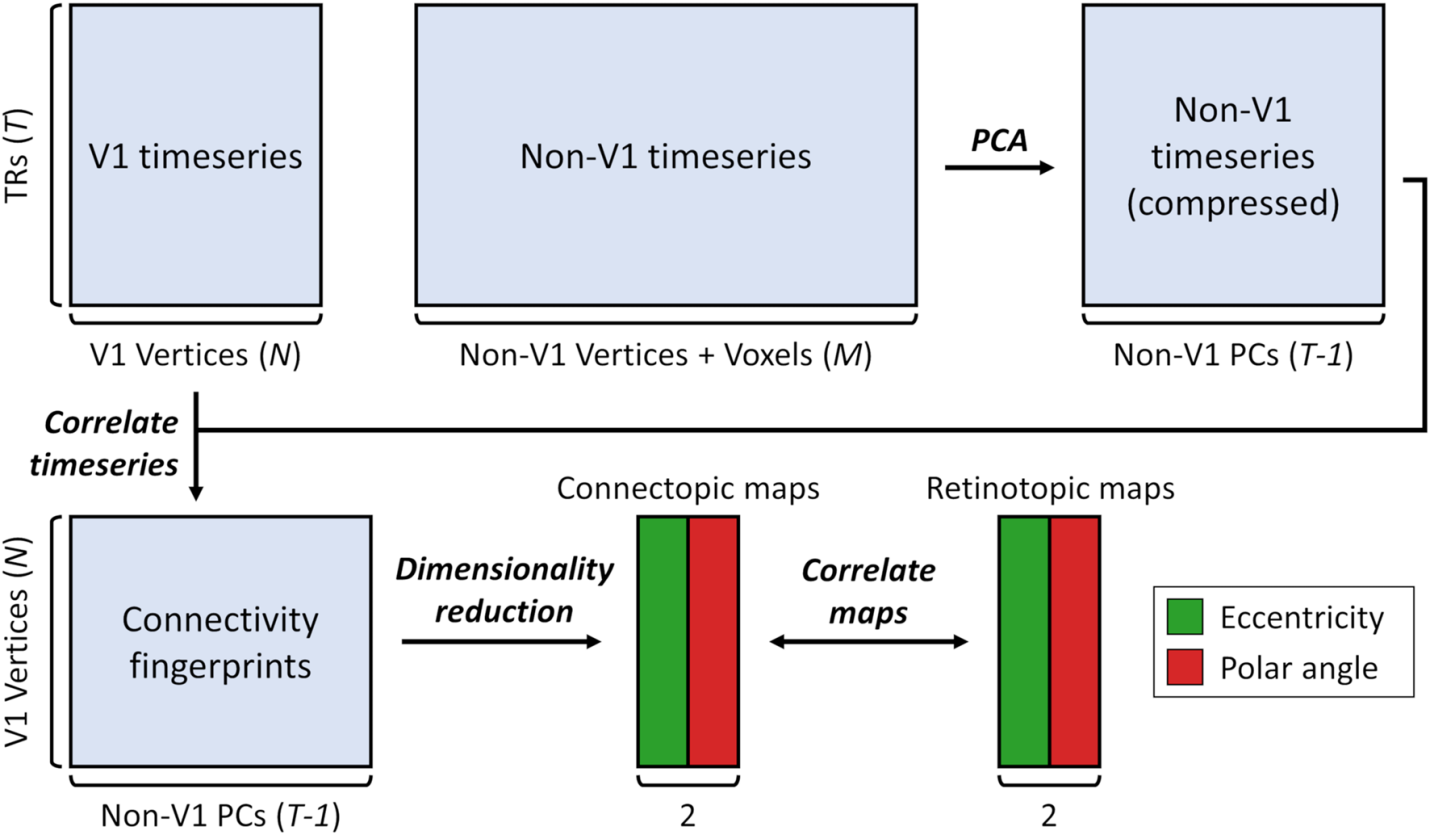
Connectopic mapping pipeline. Functional MRI timeseries, comprising *T* timepoints, are split between the *N* vertices within V1 versus the *M* cortical vertices and subcortical voxels outside V1. The non-V1 timeseries are reduced in dimensionality via a lossless principal component analysis, retaining one fewer components than the number of timepoints (*T-1*). The V1 and compressed non-V1 timeseries are then correlated to derive the connectivity space. Dimensionality reduction is applied to this space and two components are retained, yielding the connectopic maps which are expected to correspond to the eccentricity and polar angle maps respectively. Prediction accuracy is assessed by correlating the connectopic maps with retinotopic maps estimated by traditional visual field mapping.

Next, we performed connectopic mapping within each V1 ROI in each subject, adapting the methods of Haak and colleagues^22^. The analysis pipeline is illustrated in Fig. 1. First, the movie-watching data were split into odd and even scan runs to allow cross-validated parameter selection for the dimensionality reduction algorithms (where necessary). For each data split, timeseries were concatenated over scan runs yielding approximately 1 h of data per split. We extracted the timeseries within all subcortical voxels and cortical surface vertices. These were then split into those surface vertices within the V1 ROI versus all surface vertices and subcortical voxels outside the ROI. Because the number of the non-ROI vertices and voxels exceeds the number of timepoints, we losslessly compressed this data via principal component analysis, retaining one fewer components than the number of timepoints so that 100% of the variance remained explained. This amounts to rotating the samples within the feature space and removing the unused dimensions, and aids the computational tractability of later processing stages. The timeseries were then correlated between all pairwise combinations of V1 vertices and non-V1 principal components. This yielded a matrix of connectivity fingerprints representing the functional connectivity profile of each V1 vertex.

**Figure 2:**
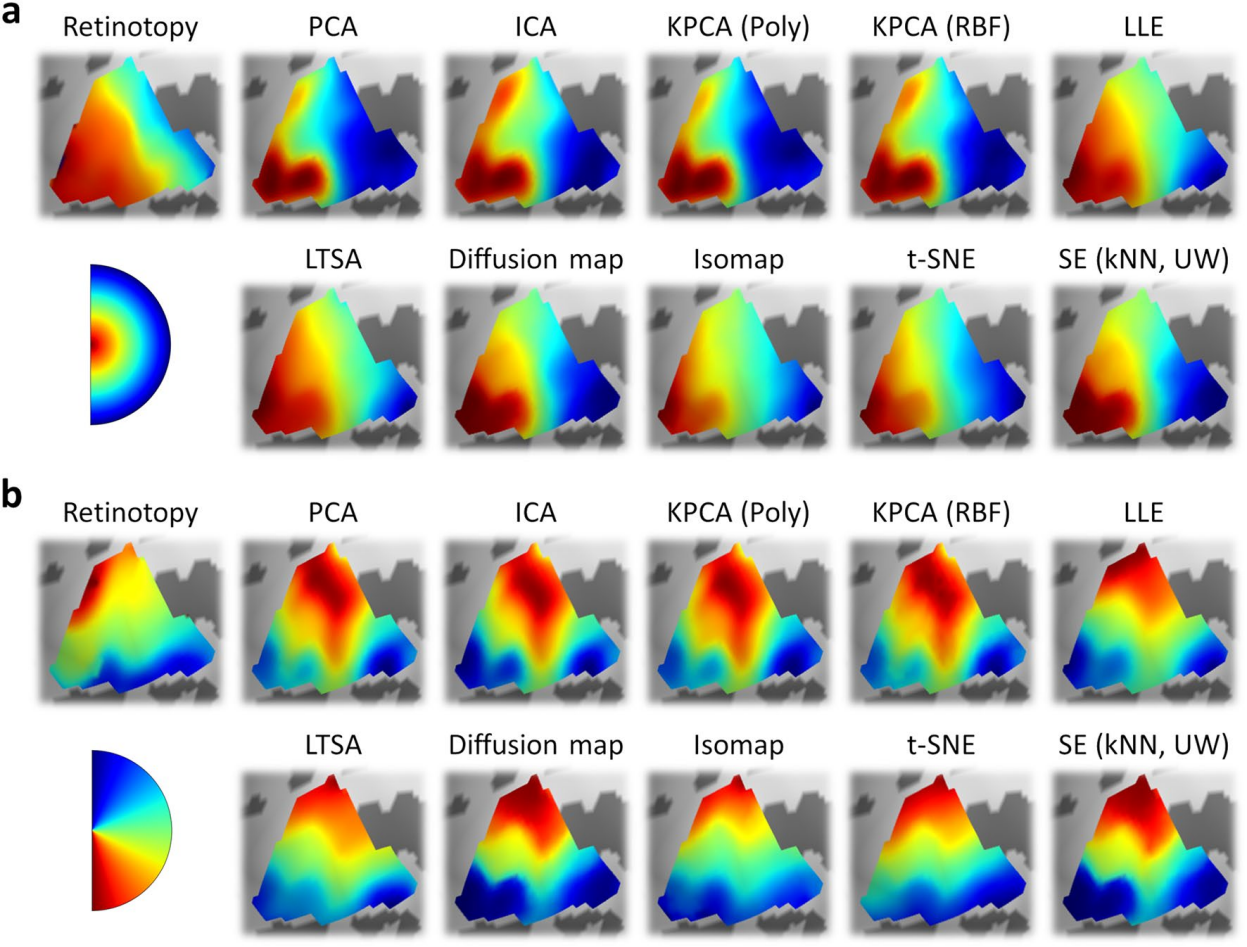
Experiment 1: Retinotopic and connectopic maps of (**a**) eccentricity and (**b**) polar angle in the left hemisphere of an example subject. The top-left plot in each panel illustrates retinotopic maps measured by traditional visual field mapping techniques. The remaining plots illustrate maps reconstructed from connectivity patterns measured during movie-watching. Spectral embedding is illustrated for the unweighted nearest neighbour variant.

The intuition of connectopic mapping is that functional gradients within the ROI correspond to principal dimensions of variation within the connectivity space. Gradients can be reconstructed by embedding the high-dimensional connectivity fingerprints into a lower-dimensional space and extracting the initial components. Here, we retained the first two components, which are expected to correspond to the eccentricity and polar angle maps. Where necessary, parameter selection for a given algorithm was performed via cross-validation using a Bayesian optimisation routine to maximise the prediction accuracy. Figure 2 illustrates retinotopic and connectopic maps in an example subject. All of the dimensionality reduction techniques captured a component following an anterior–posterior gradient which corresponded well to the retinotopic eccentricity map (Fig. 2a), as well as a component following an inferior-superior gradient which corresponded to the retinotopic polar angle map (Fig. 2b).

**Figure 3:**
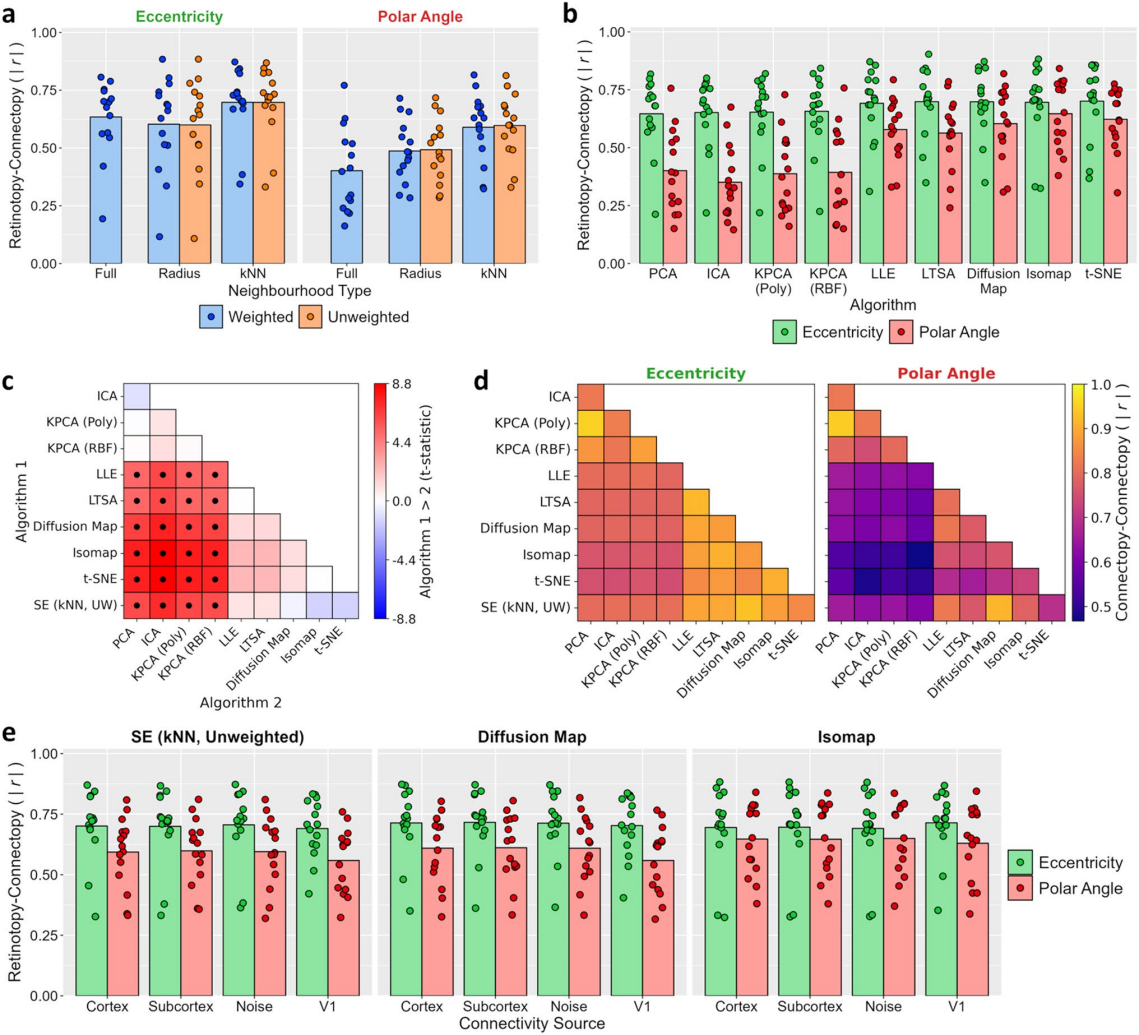
Experiment 1 results. (**a**) Prediction accuracies for all variants of spectral embedding, measured by absolute correlations between retinotopic and connectopic maps. Dot markers indicate per-subject means and bars indicate group means. (**b**) Prediction accuracies for other algorithms. (**c**) Post-hoc Tukey contrasts of prediction accuracies between algorithms. For spectral embedding, the unweighted nearest neighbour variant is selected. Dot markers indicate significant contrasts (*p* < 0.05). (**d**) Correlations of connectopic maps between algorithms. (**e**) Prediction accuracies for unweighted nearest neighbour variant of spectral embedding, diffusion map, and Isomap algorithms when varying the source of the connectivity estimates: V1 timeseries are correlated with timeseries taken from just non-V1 cortical vertices, just subcortical voxels, non-V1 cortical vertices plus subcortical voxels substituted with Gaussian noise, or the same V1 timeseries.

We next tested the prediction accuracies of each algorithm by correlating the connectopic maps estimated from the movie-watching with the ground-truth retinotopic maps estimated from the visual field mapping (Fig. 3). Because there is a sign ambiguity in the connectopic maps we took the absolute correlation values. We first considered the spectral embedding algorithm, which has previously been shown to reconstruct gradients in sensory regions including primary visual cortex^22^. We ran variants based on both weighted and unweighted versions of both radius and nearest neighbours (kNN) graphs, as well as a weighted fully-connected graph (see Methods). The resulting prediction accuracies for each variant are illustrated in Fig. 3a. Accuracies appeared highest for the nearest neighbour approach, and did not appear substantially different between weighted and unweighted variants. In all cases, correlations were significantly greater than zero (one-sample t-tests; all *p* < 0.001). To compare between the variants, we entered the correlations into a two-way repeated-measures ANOVA with factors of map (eccentricity, polar angle) and graph type. This revealed a significant main effect of map type due to overall higher correlations for eccentricity than polar angle (F(1, 14) = 8.20, *p* = 0.013, $\eta_{P}^{2}$ = 0.37, $\eta_{G}^{2}$ = 0.18). There was also a significant main effect of graph type (F(1.59, 22.20) = 16.18, *p* < 0.001, $\eta_{P}^{2}$ = 0.54, $\eta_{G}^{2}$ = 0.11); post-hoc Tukey contrasts revealed that correlations for the nearest neighbour variants were significantly higher than the radius and fully-connected variants (all *p* < 0.001), while there were no significant differences between the radius and fully-connected variants (all *p* > 0.05), nor between weighted and unweighted variants (all *p* > 0.05). Finally, there was no significant interaction (F(1.94, 27.19) = 2.43, *p* = 0.108, $\eta_{P}^{2}$ = 0.15, $\eta_{G}^{2}$ = 0.01). Thus, all variants of spectral embedding predicted the retinotopic maps above chance, but the nearest neighbour approach performed best, and there was no clear benefit to weighting the graphs.

We next considered a number of other commonly used dimensionality reduction techniques (Fig. 3b), including linear embeddings (PCA and ICA) and various other manifold learning approaches: kernel PCA, locally linear embedding (LLE), local tangent space alignment (LTSA), diffusion maps, Isomap, and t-distributed stochastic neighbourhood embedding (t-SNE). For the kernel PCA, we employed two commonly used kernels: a second-order polynomial kernel, and a radial basis function (RBF) kernel. Again, one-sample t-tests revealed that correlations with the retinotopic maps were significantly greater than zero in all cases (one-sample t-tests; all *p* < 0.001). Furthermore, paired-samples t-tests revealed significantly higher correlations for the eccentricity than the polar angle maps for all algorithms (all *p* < 0.05) except Isomap (t(14) = 1.27, *p* = 0.225) and t-SNE (t(14) = 1.66, *p* = 0.119).

Prediction accuracies generally appeared higher for the manifold learning than the linear embedding methods. To compare accuracies between algorithms, we entered the correlations into a two-way repeated measures ANOVA with factors of map and algorithm type. For spectral embedding, we selected the unweighted nearest neighbour approach as one of the better performing variants. The main effect of map type was significant (F(1, 14) = 11.00, *p* = 0.005, $\eta_{P}^{2}$ = 0.44, $\eta_{G}^{2}$ = 0.24) due to higher correlations for eccentricity than polar angle maps. There was also a significant main effect of the algorithm type (F(3.01, 42.08) = 25.23, *p* < 0.001, $\eta_{P}^{2}$ = 0.64, $\eta_{G}^{2}$ = 0.16); post-hoc Tukey contrasts (Fig. 3c) revealed that prediction accuracies were significantly lower for the PCA, ICA, and kernel PCA algorithms than for the other manifold learning techniques (all *p* < 0.001). There was also an interaction between the map and algorithm type (F(2.47, 34.61) = 6.61, *p* = 0.002, $\eta_{P}^{2}$ = 0.32, $\eta_{G}^{2}$ = 0.05) reflecting a greater difference between algorithms for the polar angle than for the eccentricity maps. We also assessed the relative similarities of the algorithms by correlating the connectopic maps between them (Fig. 3d). Similar to the prediction accuracies, this indicated that the algorithms fell into two broad groups: the linear embeddings and kernel PCAs produced relatively similar gradients to one another, and the remaining manifold learning methods also appeared similar to one another, but gradients were less similar between the two groups. In summary, the manifold learning approaches generally outperformed the linear approaches (PCA and ICA), especially when predicting the polar angle maps. Both variants of the kernel PCA (polynomial and RBF) performed more poorly and did not differ significantly from the linear PCA, suggesting that neither of these kernels were able to sufficiently capture the non-linearities in the data.

In all of the previous analyses, the connectivity fingerprints were estimated between V1 vertices and all cortical vertices plus subcortical voxels throughout the rest of the brain. To test how the source of connectivity information affects the estimated connectopic maps, we recalculated the connectivity fingerprints by correlating the V1 timeseries with the timeseries from either just the cortical vertices or just the subcortical voxels. We repeated our analyses with the spectral embedding (unweighted nearest neighbour), diffusion map, and Isomap algorithms based on these new fingerprints (Fig. 3e). Prediction accuracies remained high (one-sample t-tests: all *p* < 0.001) and did not appear substantially different to the original analyses. This was surprising as we expected the connectopic map with the cortical vertices to be a better predictor of the functional organisation.

A possible explanation for this result is that the key factor in the connectopic mapping is the topographic organisation of the functional responses within the region of interest, rather than connectivity with the rest of the brain. To test this possibility, we performed two further variants of our analyses. Firstly, we estimated connectivity fingerprints using signals from all cortical vertices plus subcortical voxels (as per the main analyses), but first substituted the non-V1 timeseries with normally distributed noise matched in mean and variance to the original timeseries. Secondly, we estimated within-ROI fingerprints by correlating each of the V1 timeseries with each other, entirely omitting timeseries outside of V1. Again, both variants produced similar results to the original analyses (Fig. 3e). In both cases, prediction accuracies remained high (one-sample t-tests: all *p* < 0.001). We entered the correlations into a three-way repeated-measures ANOVA with factors of map and algorithm type, and connectivity source (cortex + subcortex, cortex, subcortex, noise, and within-V1). This revealed significant main effects of map type (F(1, 14) = 6.06, *p* = 0.027, $\eta_{P}^{2}$ = 0.30, $\eta_{G}^{2}$ = 0.13) and algorithm type (F(1.14, 16.01) = 8.31, *p* = 0.008, $\eta_{P}^{2}$ = 0.37, $\eta_{G}^{2}$ = 0.01); post-hoc Tukey contrasts revealed overall higher correlations for Isomap than spectral embedding (*p* = 0.001) and diffusion maps (*p* = 0.028). However, the main effect of the connectivity source was not significant (F(1.20, 16.87) = 3.01, *p* = 0.095, $\eta_{P}^{2}$ = 0.18, $\eta_{G}^{2}$ < 0.01). Finally, there were significant interactions between the map and algorithm type (F(1.20, 16.79) = 6.35, *p* = 0.018, $\eta_{P}^{2}$ = 0.31, $\eta_{G}^{2}$ = 0.01) and between the algorithm type and connectivity source (F(2.72, 38.04) = 5.92, *p* = 0.003, $\eta_{P}^{2}$ = 0.30, $\eta_{G}^{2}$ < 0.01). In summary, varying the source of the connectivity information had minimal impact on the connectopic maps and in all cases prediction accuracies remained high. This suggests the principal source of information in ‘connectopic’ mapping is the functional topography within the ROI.

Finally, we tested the stability of the connectopic mapping algorithms by correlating the connectopic maps between the two cross-validation splits (Supplementary Fig. S1). The split-half correlations appeared high in all cases, indicating good internal reliability within each algorithm and suggesting that the connectopic maps are at least partially independent from the precise content of the movie stimulus.

### Experiment 2

Our first experiment reported exploratory analyses of one naturalistic imaging dataset. To determine the robustness of these results we next conducted a preregistered replication experiment using a larger 7T MRI dataset obtained from the Human Connectome Project^35^. This additionally allowed us to compare connectopic mapping between connectivity measured during both rest and movie watching. The dataset comprised 174 subjects each with approximately one hour of resting-state and movie-watching data in addition to visual field mapping scans.

**Figure 4:**
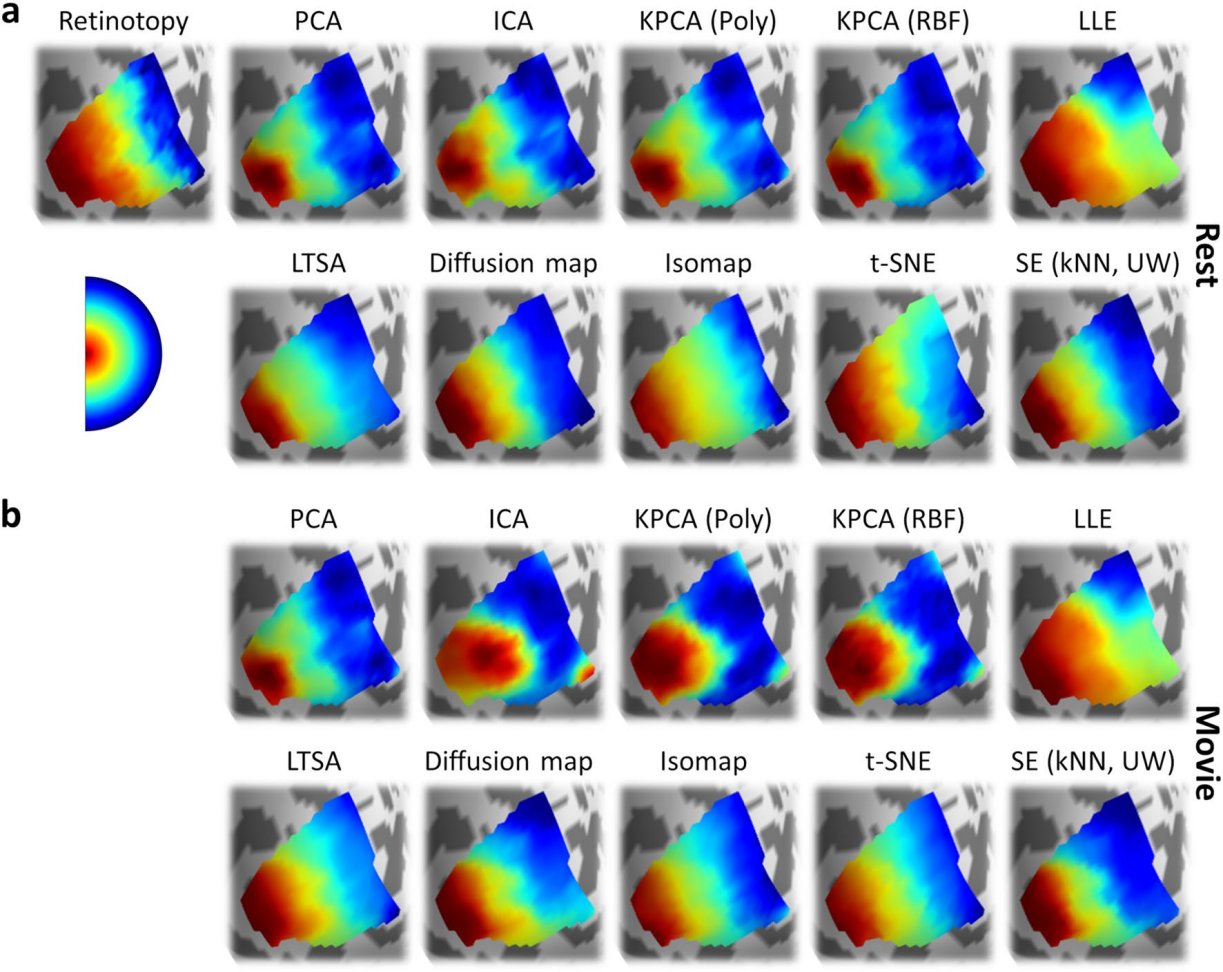
Experiment 2: Retinotopic and connectopic eccentricity maps in the left hemisphere of an example subject. (**a**) Retinotopic maps (top-left plot) and connectopic maps reconstructed from resting-state data (remaining plots). (**b**) Connectopic maps reconstructed from movie-watching data. Spectral embedding is illustrated for the unweighted nearest neighbour variant.

**Figure 5:**
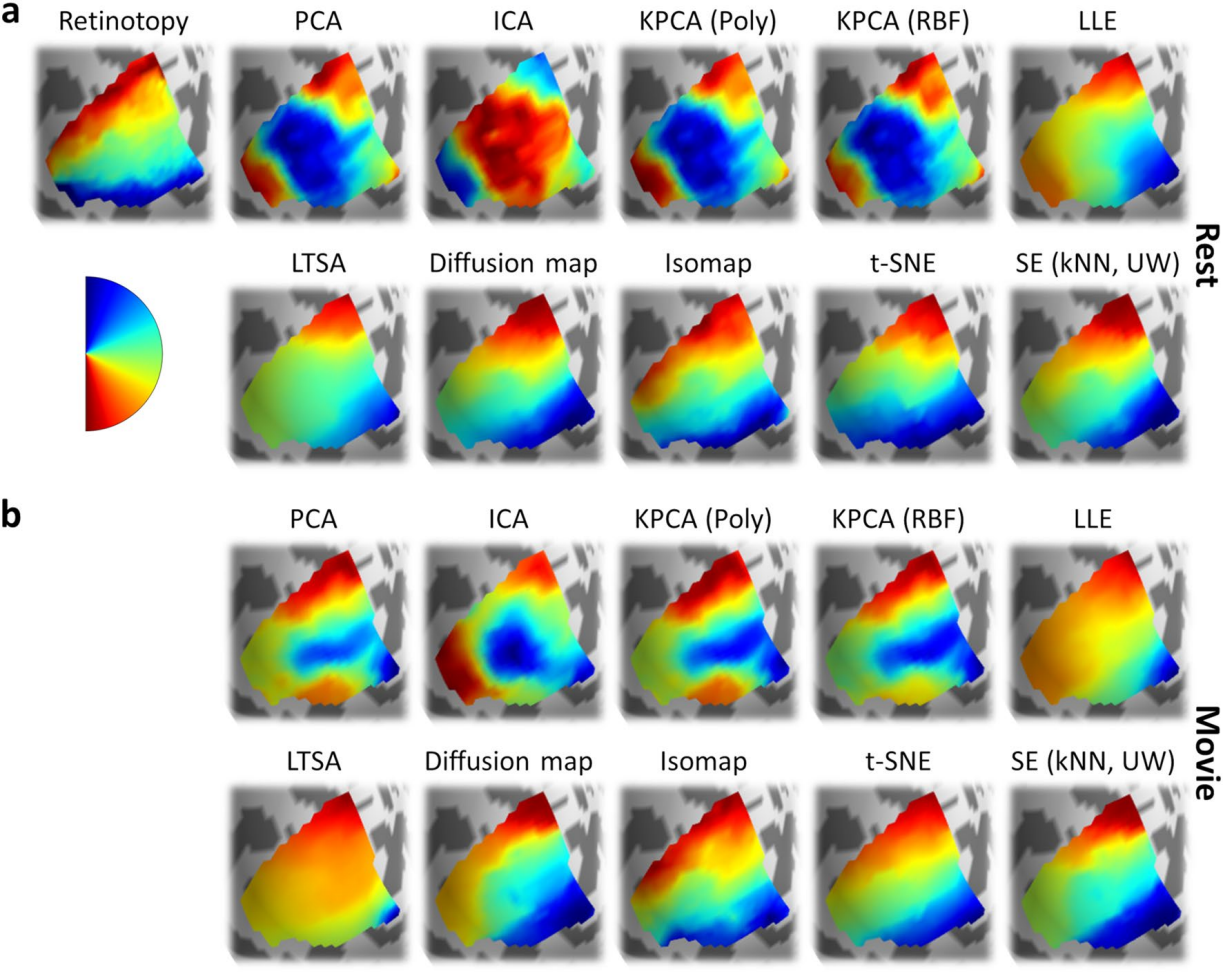
Experiment 2: Retinotopic and connectopic polar angle maps in the left hemisphere of an example subject. (**a**) Retinotopic maps (top-left plot) and connectopic maps reconstructed from resting-state data (remaining plots). (**b**) Connectopic maps reconstructed from movie-watching data. Spectral embedding is illustrated for the unweighted nearest neighbour variant.

We first extracted visual field maps in each subject via population receptive field modelling^18^. As before, these maps were used to define individualised V1 ROIs as well as forming ground-truth estimates to compare the connectopic maps against. We then performed connectopic mapping in each subject, following the same methods as for Experiment 1. Retinotopic and connectopic maps of eccentricity (Fig. 4) and polar angle (Fig. 5) are shown for an example subject during the resting-state and movie-watching tasks. All of the dimensionality reduction techniques captured an anterior–posterior gradient which corresponded well to the retinotopic eccentricity map (Fig. 4), and most captured an inferior-superior gradient corresponding to the retinotopic polar angle map (Fig. 5).

**Figure 6:**
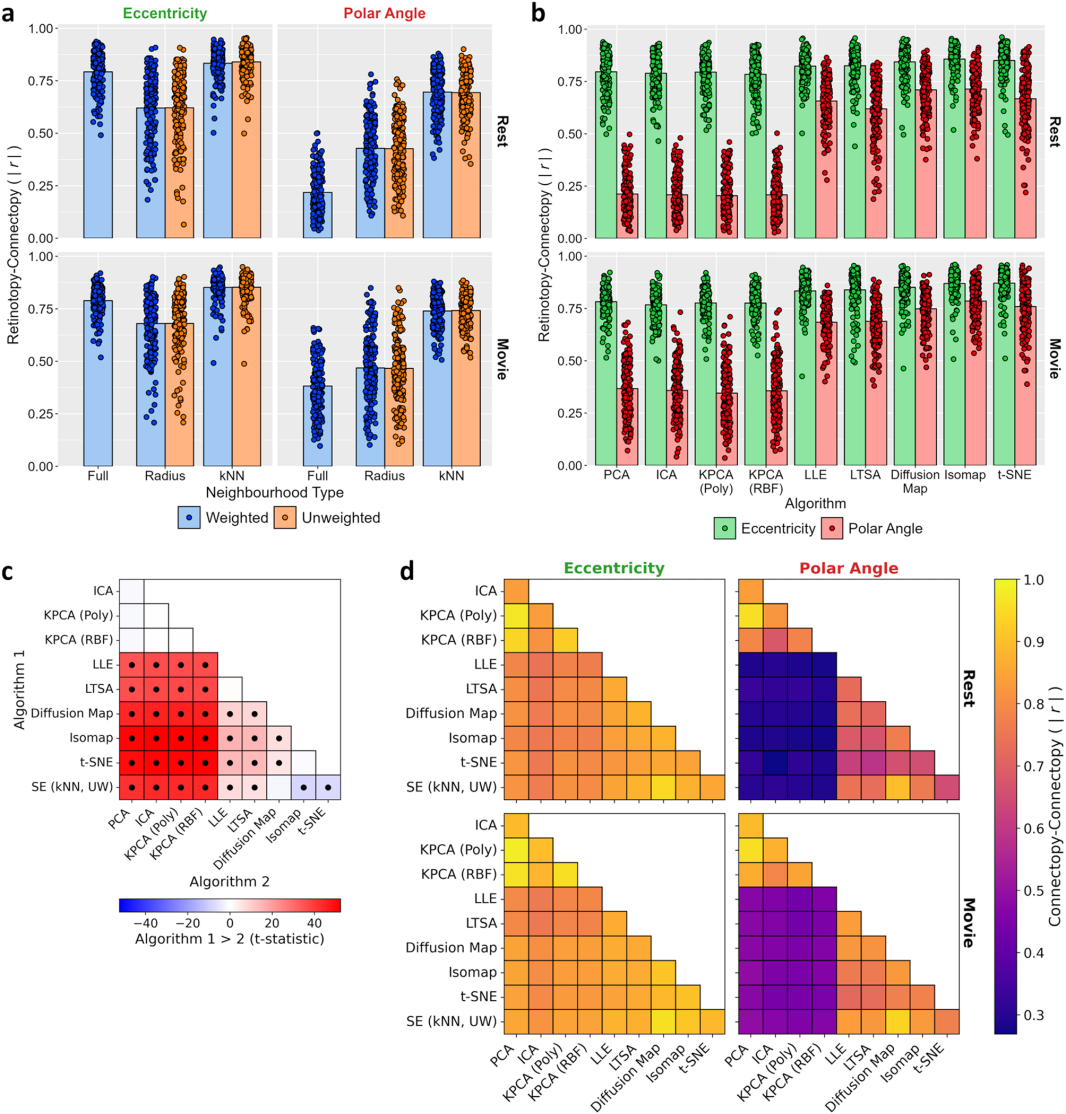
Experiment 2 main results. (**a**) Prediction accuracies for all variants of spectral embedding, measured by absolute correlations between retinotopic and connectopic maps. Dot markers indicate per-subject means, and bars indicate group means. Resting-state and movie-watching results are illustrated on top and bottom rows respectively. (**b**) Prediction accuracies for other algorithms. (**c**) Post-hoc Tukey contrasts of prediction accuracies between algorithms. For spectral embedding, the unweighted nearest neighbour variant is selected. Dot markers indicate significant contrasts (*p* < 0.05). (**d**) Correlations of connectopic maps between algorithms.

We next tested the prediction accuracies of each algorithm by taking the absolute correlations between the retinotopic and connectopic maps (Fig. 6). We first considered the spectral embedding algorithm (Fig. 6a). As per Experiment 1, accuracies appeared highest for the nearest neighbour approach and were minimally affected by weighting the graphs. In addition, accuracies appeared higher for movie-watching than resting-state estimates, particularly for polar angle maps. In all cases, correlations were significantly greater than zero (one-sample t-tests; all *p* < 0.001). To compare between the variants, we entered the correlations into a three-way repeated-measures ANOVA with factors of map type (eccentricity, polar angle), graph type, and task (resting-state, movie-watching). This revealed a significant main effect of map type reflecting overall higher correlations for eccentricity than polar angle maps (F(1, 173) = 883.07, *p* < 0.001, $\eta_{P}^{2}$ = 0.84, $\eta_{G}^{2}$ = 0.52). There was also a significant main effect of graph type (F(1.96, 339.30) = 1068.47, *p* < 0.001, $\eta_{P}^{2}$ = 0.86, $\eta_{G}^{2}$ = 0.50); post-hoc Tukey contrasts revealed that correlations were significantly higher for the nearest neighbour than the radius or fully-connected variants (all *p* < 0.001), and the fully-connected variant also outperformed both the weighted (*p* = 0.049) and unweighted (*p* = 0.035) radius neighbour variants, while there were no significant differences between weighted and unweighted radius neighbourhood or nearest neighbour variants (all *p* > 0.05). The main effect of task was also significant, reflecting higher correlations for movie-watching than resting-state datasets (F(1, 173) = 55.89, *p* < 0.001, $\eta_{P}^{2}$ = 0.24, $\eta_{G}^{2}$ = 0.03). There were significant two-way interactions of map by graph type (F(2.14, 369.87) = 441.75, *p* < 0.001, $\eta_{P}^{2}$ = 0.72, $\eta_{G}^{2}$ = 0.16) and map type by task (F(1, 173) = 14.81, *p* < 0.001, $\eta_{P}^{2}$ = 0.08, $\eta_{G}^{2}$ < 0.01) reflecting greater differences between both graph types and tasks for polar angle than eccentricity maps. The graph type by task interaction was not significant (F(1.76, 304.31) = 1.34, *p* = 0.263, $\eta_{P}^{2}$ = 0.01, $\eta_{G}^{2}$ < 0.01). Finally, there was also a significant three-way map type by graph type by task interaction (F(2.02, 349.57) = 38.02, *p* < 0.001, $\eta_{P}^{2}$ = 0.18, $\eta_{G}^{2}$ = 0.01).

We next considered the other connectopic mapping algorithms (Fig. 6b). As before, prediction accuracies appeared higher for the manifold learning than the linear embedding methods, and higher for movie-watching than resting-state estimates. One-sample t-tests revealed that correlations with the retinotopic maps were significantly greater than zero in all cases (one-sample t-tests; all *p* < 0.001). We further entered the correlations for each algorithm into a series of two-way repeated-measures ANOVAs with factors of map type and task ‒ the results of these analyses are listed in Table 1. All algorithms showed a significant main effect of map type due to higher correlations for eccentricity than polar angle maps, as well as a significant main effect of task due to higher correlations for movie-watching than resting-state datasets. Additionally, all algorithms except LLE and LTSA showed a significant map type by task interaction.

**Table 1:** Experiment 2: Two-way repeated-measures ANOVAs of prediction accuracies with factors for map type (eccentricity, polar angle) and task (rest, movie-watching) for all connectopic mapping algorithms except spectral embedding.

Algorithm	Factor	F	DoF	*p*	$\eta_{P}^{2}$	$\eta_{G}^{2}$
PCA	Map type	2381.73	1, 173	** < 0.001**	0.93	0.84
	Task	27.91	1, 173	** < 0.001**	0.14	0.02
	Interaction	129.24	1, 173	** < 0.001**	0.43	0.11
ICA	Map type	2522.95	1, 173	** < 0.001**	0.94	0.84
	Task	16.13	1, 173	** < 0.001**	0.09	0.01
	Interaction	133.63	1, 173	** < 0.001**	0.44	0.13
KPCA (Poly)	Map type	2553.89	1, 173	** < 0.001**	0.94	0.84
	Task	16.48	1, 173	** < 0.001**	0.09	0.01
	Interaction	113.19	1, 173	** < 0.001**	0.40	0.11
KPCA (RBF)	Map type	2516.78	1, 173	** < 0.001**	0.94	0.83
	Task	32.93	1, 173	** < 0.001**	0.16	0.03
	Interaction	97.49	1, 173	** < 0.001**	0.36	0.10
LLE	Map type	686.93	1, 173	** < 0.001**	0.80	0.53
	Task	6.51	1, 173	**0.012**	0.04	0.01
	Interaction	0.07	1, 173	0.797	< 0.01	< 0.01
LTSA	Map type	544.03	1, 173	** < 0.001**	0.76	0.51
	Task	36.86	1, 173	** < 0.001**	0.18	0.03
	Interaction	3.39	1, 173	0.067	0.02	< 0.01
Diffusion Map	Map type	451.98	1, 173	** < 0.001**	0.72	0.41
	Task	12.56	1, 173	** < 0.001**	0.07	0.01
	Interaction	9.63	1, 173	**0.002**	0.05	< 0.01
Isomap	Map type	424.30	1, 173	** < 0.001**	0.71	0.38
	Task	50.07	1, 173	** < 0.001**	0.22	0.05
	Interaction	64.41	1, 173	** < 0.001**	0.27	0.02
t-SNE	Map type	373.70	1, 173	** < 0.001**	0.68	0.35
	Task	57.68	1, 173	** < 0.001**	0.25	0.07
	Interaction	27.38	1, 173	** < 0.001**	0.14	0.02

Significant *p*-values are highlighted in bold.

To compare prediction accuracies between algorithms, we further entered the correlations into a three-way repeated measures ANOVA with factors of map type, task, and algorithm type. For spectral embedding, we selected the unweighted nearest neighbour approach as one of the better performing variants. There were significant main effects of map type (F(1, 173) = 1614.28, *p* < 0.001, $\eta_{P}^{2}$ = 0.90, $\eta_{G}^{2}$ = 0.65) reflecting higher correlations for eccentricity than polar angle maps, of task (F(1, 173) = 87.33, *p* < 0.001, $\eta_{P}^{2}$ = 0.34, $\eta_{G}^{2}$ = 0.03) reflecting higher correlations for movie-watching than resting-state, and of algorithm type (F(3.40, 587.99) = 1084.96, *p* < 0.001, $\eta_{P}^{2}$ = 0.86, $\eta_{G}^{2}$ = 0.55). Post-hoc Tukey contrasts (Fig. 6c) revealed that Isomap and t-SNE outperformed spectral embedding and diffusion maps, which in turn outperformed LLE and LTSA, which in turn outperformed PCA, ICA, and the kernel PCAs (all *p* < 0.001). There were no significant differences between Isomap and t-SNE, or between spectral embedding and diffusion maps, or between LLE and LTSA, or between PCA, ICA, and the kernel PCAs (all *p* > 0.05). There were significant two-way interactions of map type by task (F(1, 173) = 111.59, *p* < 0.001, $\eta_{P}^{2}$ = 0.39, $\eta_{G}^{2}$ = 0.03) and of map by algorithm type (F(3.95, 682.74) = 502.74, *p* < 0.001, $\eta_{P}^{2}$ = 0.74, $\eta_{G}^{2}$ = 0.23) because the difference between both tasks and algorithms was greater for polar angle than eccentricity maps, and a significant task by algorithm type interaction (F(5.23, 905.24) = 8.84, *p* < 0.001, $\eta_{P}^{2}$ = 0.05, $\eta_{G}^{2}$ = 0.01) because the difference between tasks was reduced for better performing algorithms. Finally, there was a significant three-way map type by task by algorithm type interaction (F(4.30, 744.17) = 39.58, *p* < 0.001, $\eta_{P}^{2}$ = 0.19, $\eta_{G}^{2}$ = 0.02).

We also tested the consistency of the connectopic maps between algorithms by correlating the maps (Fig. 6d). As before, the linear embeddings and kernel PCAs produced relatively similar gradients, and the remaining manifold learning methods also yielded similar gradients, but gradients appeared less similar between these two groups. Additionally, gradients appeared more consistent when based on movie-watching than on resting-state data. In summary, the manifold learning algorithms outperformed the linear embeddings and both kernel PCAs (especially when predicting the polar angle maps), with Isomap and t-SNE performing best, and performance was better when connectivity was estimated from movie-watching than resting-state data.

**Figure 7:**
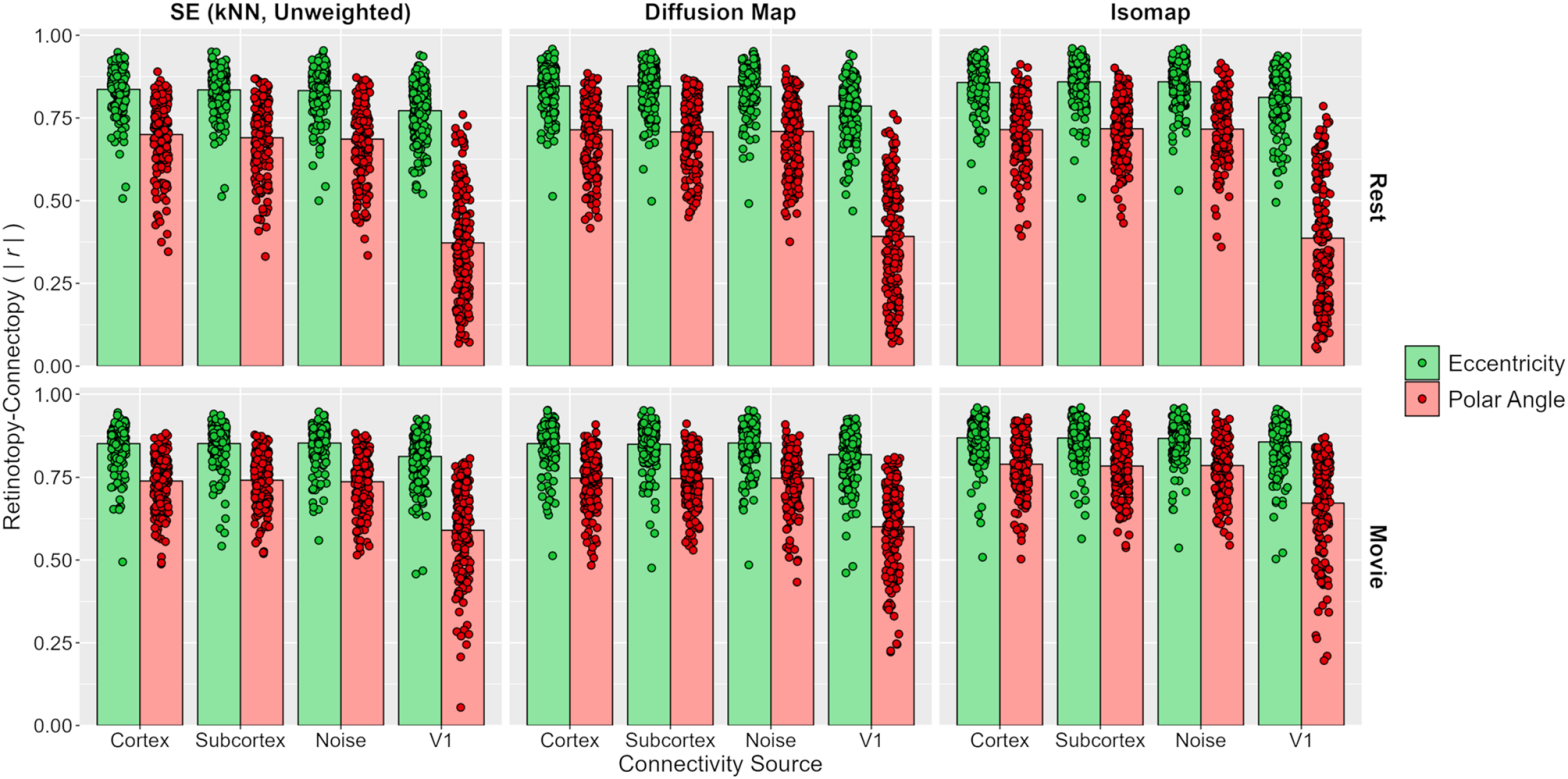
Experiment 2: Prediction accuracies for unweighted nearest neighbour variant of spectral embedding, diffusion map, and Isomap algorithms when varying the source of the connectivity fingerprints. V1 timeseries are correlated with timeseries taken from just non-V1 cortical grayordinates, just subcortical grayordinates, non-V1 cortical plus subcortical grayordinates substituted with Gaussian noise, or the same V1 timeseries. Resting-state and movie-watching results are illustrated on top and bottom rows respectively.

We next tested how the source of the connectivity information affects the connectopic mapping. We recalculated the connectivity fingerprints by correlating the V1 timeseries with the timeseries from either just the cortical vertices, just the subcortical voxels, the cortical vertices and subcortical voxels substituted with Gaussian noise, or the same V1 timeseries. We repeated the connectopic mapping with the spectral embedding (unweighted nearest neighbour), diffusion map, and Isomap algorithms using these new fingerprints (Fig. 7). Similar to Experiment 1, the pattern of results appeared largely similar across the different connectivity sources. As before, all prediction accuracies remained high (one-sample t-tests: all *p* < 0.001). We entered the correlations into a four-way repeated-measures ANOVA with factors of map type, task, algorithm type, and connectivity source (cortex + subcortex, cortex, subcortex, noise, and within-V1). This revealed significant main effects of map type (F(1, 173) = 745.28, *p* < 0.001, $\eta_{P}^{2}$ = 0.81, $\eta_{G}^{2}$ = 0.47), task (F(1, 173) = 132.43, *p* < 0.001, $\eta_{P}^{2}$ = 0.43, $\eta_{G}^{2}$ = 0.05), and algorithm type (F(1.42, 245.83) = 190.49, *p* < 0.001, $\eta_{P}^{2}$ = 0.52, $\eta_{G}^{2}$ = 0.04); post-hoc Tukey contrasts indicated correlations were significantly higher for Isomap than diffusion maps, and both were higher than spectral embedding (all *p* < 0.001). Unlike Experiment 1, the main effect of connectivity source was now also significant (F(1.22, 211.02) = 597.45, *p* < 0.001, $\eta_{P}^{2}$ = 0.78, $\eta_{G}^{2}$ = 0.20); post-hoc Tukey contrasts indicated this was due to significantly lower correlations for the within-V1 analyses than the other sources (all *p* < 0.001), while the remaining sources did not differ significantly (all *p* > 0.05). Note that this result may reflect differences in the pre-processing pipeline for the within-V1 analyses compared to other sources (see Unregistered Analyses). Finally, all interactions were significant (all *p* < 0.001) except for the two-way map type by task interaction (*p* = 0.443) and the four-way map type by task by algorithm type by connectivity source interaction (*p* = 0.392).

Finally, we conducted two further analyses to examine the stability of the connectopic mapping algorithms. Firstly, we investigated the split-half reliability by correlating the connectopic maps between the two cross-validation splits. The correlations appeared high in all cases (Supplementary Fig. S2). Secondly, we correlated the connectopic maps between the cross-validation splits *and* tasks (e.g. correlating maps for odd resting-state runs with even movie-watching runs). Again, the correlations appeared high in all cases (Supplementary Fig. S3). This indicates good internal reliability within each algorithm, that the connectopic maps are partially independent of the precise content of the movie stimulus, and that the maps generalise well between resting-state and movie-watching tasks.

#### Unregistered analyses

**Figure 8:**
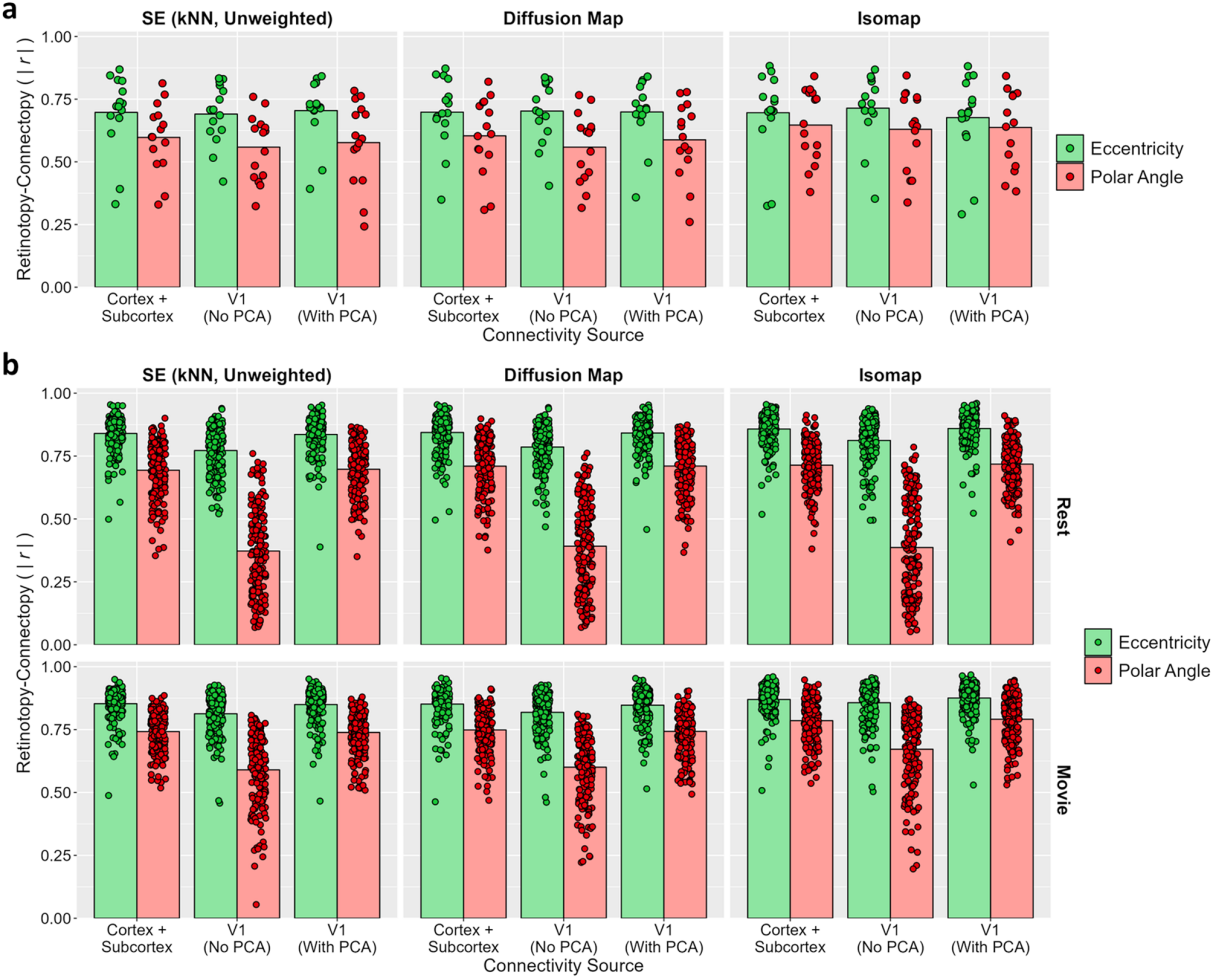
Exploratory analyses testing effect of applying lossless PCA compression to timeseries prior to calculating connectivity fingerprints for within-V1 analyses. Plots illustrate prediction accuracies, measured by absolute correlations between retinotopic and connectopic maps, for the unweighted nearest neighbour variant of spectral embedding, diffusion map, and Isomap algorithms. Results are illustrated for (**a**) Experiment 1 and (**b**) Experiment 2: resting-state and movie-watching results are illustrated on top and bottom rows respectively. Left and middle bar groups duplicate results from Figs. 3, 6, and 7: V1 timeseries are correlated with timeseries taken from non-V1 cortical vertices and subcortical voxels (following PCA compression) or the same V1 timeseries (without PCA compression). Right bar groups illustrate accuracies when V1 timeseries are correlated with principal components derived from the same V1 timeseries.

In our preregistered analyses, we observed an apparent reduction in prediction accuracies for the within-V1 analyses compared to those of other connectivity sources. One key difference between the within-V1 analyses and those of the other sources is that we omit the PCA transformation of the timeseries prior to calculating the connectivity fingerprints (Fig. 1). This is because the number of timepoints already exceeds the number of vertices within the ROI and hence the dimensionality cannot be reduced further without losing information. Nevertheless, it is possible that the reduction in prediction accuracy for the within-V1 analyses relative to other sources reflects the omission of this processing stage rather than the connectivity information represented within the region. To test this possibility, we conducted exploratory analyses repeating the within-V1 analyses but including the PCA transformation stage, such that the V1 timeseries were now correlated with principal components derived from those same timeseries. The results of these analyses are shown in Fig. 8. While the inclusion of the PCA transformation had minimal effect on the StudyForrest data (Experiment 1), including it for the HCP data (Experiment 2) recovered performance to levels comparable with the main analyses. This suggests the apparent reduction in performance for the within-V1 analyses mostly reflects methodological differences in the processing pipeline compared to the other neural sources. In general, as per Experiment 1, varying the connectivity source had minimal effect on the connectopic mapping, suggesting that the functional topography within the region is the key factor.

## Discussion

This study aimed to determine how connectopic mapping predicts functional gradients in primary visual cortex by benchmarking reconstructions against ground-truth estimates of retinotopic maps measured by visual field mapping. We reported results from an exploratory analysis using data from natural viewing (movie-watching), followed by a preregistered replication experiment using both resting-state and natural viewing data. In both experiments, connectopic mapping accurately reconstructed retinotopic maps across subjects. More advanced manifold learning methods outperformed simpler methods including linear embeddings (especially for polar angle maps), with Isomap and t-SNE performing best. Additionally, prediction accuracy was better for eccentricity than polar angle maps, and better when using connectivity estimated during natural viewing than during rest. Varying the neural source of the connectivity estimates had minimal impact on the connectopic mapping, suggesting the key factor is the functional topography within the region of interest.

Previously, Haak and colleagues demonstrated that connectopic mapping (via spectral embedding) using resting-state data reconstructed gradients resembling retinotopic maps in primary visual cortex^22^. We replicated these findings, and further compared the connectopic maps against ground-truth estimates of the retinotopic maps measured by traditional visual field mapping techniques. We confirmed that connectopic maps reconstructed from both resting-state and movie-watching data accurately predicted retinotopic maps across subjects. The significance of these findings is that they provide validated methods for exploring gradients in other brain regions, and indeed connectopic mapping techniques have been applied to other regions including motor cortex^22^, the striatum^26^, and the hippocampus^27^. Such approaches require that a region of interest is first defined to perform the connectopic mapping within. This could, for instance, be done functionally (e.g. via visual field mapping or a functional localiser), or by using an atlas- or parcellation-based approach. Connectopic mapping has also been used to recover coarser scale gradients over the whole brain^16,25^, although ascertaining the accuracy of such gradients may be more challenging as the ground-truth is often unknown. We obtained remarkably consistent results between both experiments, suggesting that connectopic mapping is robust to differences in stimuli and tasks (e.g. continuous movie sequences in Experiment 1, compared to rest or compilations of short movie clips in Experiment 2), data acquisition parameters (e.g. 3T vs. 7T, single- vs. multi-band, etc.), and pre-processing pipelines. This approach offers the possibility of deriving functional gradients throughout the brain, including in regions where the organising principles are currently poorly understood or unknown. In regions where functional gradients have previously been described, they are typically observed to be topographically organised such that there is a gradual change in response properties which is mapped smoothly across a brain region. Using connectopic mapping, it will therefore be possible to determine if topographic maps are a ubiquitous organising principle throughout the brain^1,15^.

Previous studies employing connectopic mapping have exclusively measured connectivity in the brain at rest. Although resting-state is a common choice, it has been criticised as an unnatural cognitive state^30^. Here, we compared the performance of connectopic mapping when estimating connectivity at rest or during natural viewing (movie-watching). Not only did connectopic mapping perform well using movie-watching data, but prediction accuracies exceeded those obtained from resting-state. Previous studies have indicated movie-watching elicits functional connectivity that is less widespread and reduced in magnitude compared to resting-state^38–40^, likely due to stimulus driven responses disrupting spontaneous or intrinsic brain activity. However, connectivity from movie-watching may be more predictive of function in some brain regions including visual cortex^32,41^, and our results are consistent with this conclusion. Movie-watching may also offer other advantages such as improved reliability of connectivity estimates^39,42^, reproducibility of brain activity across participants and data acquisition sites^43^, and suitability for studying developmental populations^44,45^. Nevertheless, the gradients could still be accurately reconstructed from connectivity at rest, and there may be scenarios where resting-state data would be advantageous, for instance if studying visually impaired populations. Furthermore, resting-state data may be better predictive of function in non-sensory regions such as within the default mode network^32^. The spatial topography of connectivity is typically similar between rest and movie-watching^38,40^ (but see^46^), and resting-state connectivity is predictive of brain activity during movie-watching^47^, thus both approaches are likely to reveal similar aspects of functional connectivity.

One key question is the extent to which the neural source of the connectivity signals affects connectopic mapping within the region of interest. To test this, we repeated our analyses while varying the source of the connectivity signals. We first measured connectivity between vertices in V1 and either just the cortical vertices or just the subcortical voxels (as opposed to the combination of both in the main analyses). Surprisingly, this had minimal effect on the connectopic maps: prediction accuracies remained comparable to those of the main analyses. This suggests that the main driver of connectopic mapping is the functional topography within the region itself. For instance, two functionally similar voxels within the region displaying relatively similar timecourses of activation will produce relatively similar patterns of connectivity with the rest of the brain. Conversely, two functionally dissimilar voxels within the region will yield dissimilar connectivity fingerprints. Consequently, the functional topography within the region alone may prove sufficient to drive connectopic mapping. Indeed, we also found that performance was minimally affected when measuring connectivity purely within V1 or when substituting the non-V1 timeseries with random noise. A slight decrement in performance was observed for the within-V1 analyses in Experiment 2, however exploratory analyses indicated this likely resulted from omitting the lossless PCA transformation of the source timeseries prior to calculating the connectivity fingerprints. The PCA transformation generally reduces the magnitude of correlations in the connectivity fingerprints and biases the fingerprints to have stronger correlations along earlier than later dimensions, and potentially these properties may prove beneficial to the manifold learning. Regardless, our analyses tentatively suggest that a lossless PCA transformation of the source timeseries may be a useful standard processing step to include in connectopic mapping pipelines.

Previous studies have interpreted connectopic mapping as reflecting a topographic organisation of the connectivity itself^21,22^. In contrast, our results suggest a different interpretation in which the key factor is the functional topography within the target region, and the connectivity provides an index into this topography but the source of that connectivity is relatively unimportant. This is not to say that connectivity is not topographically organised, but rather that such organisation is not necessary for connectopic mapping to accurately predict functional gradients. Further investigations will be required to determine if this principle holds for other brain regions ‒ for instance, whether the source of the connectivity would be more important for mapping higher-level and/or non-sensory regions, or whether the within-region functional topography would remain sufficient.

A key aim of this study was to compare the efficacy of different dimensionality reduction techniques in performing connectopic mapping. We tested a number of commonly used and widely implemented algorithms including both non-linear manifold learning and linear embedding methods. We found that Isomap and t-SNE achieved the best prediction accuracies; there was no significant difference between these algorithms, although Isomap may be preferable as t-SNE is considerably more computationally expensive. Spectral embedding and diffusion maps (both of which have previously been applied to connectopic mapping^16,22^) performed next best and only slightly behind Isomap and t-SNE. Within spectral embedding, we observed best performance using a nearest-neighbourhood graph (as opposed to a radius-neighbourhood or fully-connected graph) and found little benefit to weighting the graph. Locally linear embedding and local tangent space alignment performed next best, and the linear embeddings (PCA and ICA) and kernel PCAs (with polynomial and radial basis function kernels) performed the worst. Thus, more advanced manifold learning methods outperformed the simpler linear embeddings suggesting some degree of non-linearity to the connectivity manifold—particularly in the case of polar angle maps.

Contrary to our findings, Haak and colleagues found relatively poor performance of the Isomap algorithm^22^. One possible reason for this discrepancy may be the selection of the neighbourhood parameter for the algorithm: we optimised this parameter to maximise the prediction accuracy, whereas Haak and colleagues set it to the minimum value that permitted a connected graph. Thus, appropriate parameter selection may be important to the success of connectopic mapping in deriving accurate gradients. Most manifold learning algorithms require some form of parameter selection. In our case the parameters were optimised based on the ground-truth estimates provided by the visual field mapping. However, if the ground-truth is unknown then appropriate parameter selection may be more challenging. Nevertheless, some heuristics may aid parameter selection. For example, one proposal for Isomap is to set the parameter to maximise the correlation between geodesic distances in the original space and Euclidean distances in the embedded space^48^. However, such heuristics may not be available or appropriate in all cases. If no better options are available, it may be preferable to simply observe how robust the connectopic maps are to changes in the parameter(s) over a range of values. An additional issue is that the connectopic maps may not emerge consistently or discretely along each component. For instance, the maps may emerge across different components over subjects, or could load on more than one component. One solution may be to apply a further linear transformation (e.g. Procrustes) after the manifold learning to optimise the alignment between the connectopic maps and some other reference maps (such as ground-truth estimates, or connectopic maps derived from a different participant or at the group level)^49^. We did not apply such an approach here as further transformation of the maps could have obscured differences between the dimensionality reduction algorithms.

In conclusion, we provide an appraisal of connectopic mapping methods for reconstructing functional gradients in primary visual cortex. We find that connectopic mapping can accurately predict retinotopic maps across subjects, with better performance for eccentricity than polar angle maps. We find that non-linear manifold learning algorithms outperform linear dimensionality reduction techniques, with Isomap and t-SNE performing especially well. Performance was best when using connectivity estimated during natural viewing, although prediction accuracies remained high using resting-state estimates too. The neural source of the connectivity estimates had minimal effect on the performance of the connectopic mapping suggesting that the key factor is the functional topography within the region of interest rather than a topography embedded within the connectivity itself. Developing standardised methods for performing connectopic mapping opens the possibility of applying this technique to explore topographic maps throughout the brain.

## Methods

### Experiment 1

Our first experiment presents exploratory analyses of movie-watching data obtained from a publicly available MRI dataset.

#### Dataset

We obtained movie-watching 3T MRI data from the publicly available StudyForrest dataset^34,36^ (https://www.studyforrest.org/). We used a subset comprising 15 participants who also have visual field mapping data^37^: S1–S6, S9, S10, and S14-S20. In brief, functional data were acquired on a 3 T Philips Achieva MRI scanner via an EPI sequence (TR = 2 s, TE = 30 ms, voxel resolution = 3 mm isotropic). The movie-watching stimulus comprised approximately 2 h of the “Forrest Gump” movie, and the visual field mapping stimulus comprised a flickering chequerboard presented within an aperture displaying expanding/contracting rings or rotating wedges. Full details are provided in^37^.

#### Pre-processing

Movie-watching and visual field mapping data were subjected to the same pre-processing pipeline. Some light pre-processing had already been applied by the StudyForrest project: this included motion correction using FSL’s MCFLIRT tool^50^, and aligning each volume to a common subject-specific reference volume shared across all runs. We then applied additional pre-processing using FSL’s FEAT v6.0^51,52^ (https://fsl.fmrib.ox.ac.uk/fsl/fslwiki/): slice-timing correction using Fourier-space time-series phase-shifting, non-brain removal^53^, grand-mean intensity normalisation of the entire 4D dataset by a single multiplicative factor, and high-pass temporal filtering (Gaussian-weighted least-squares straight line fitting with σ = 50 s). Spatial smoothing was not applied at this stage. The functional timeseries were then normalised by first converting to units of percentage signal change, and then finally regressing out both the mean ventricular and white-matter timeseries and motion parameters.

The pre-processed timeseries were co-registered to anatomical spaces. Cortical surfaces in each subject were reconstructed from T1- and T2-weighted anatomical images using Freesurfer v6.0^54^ (https://surfer.nmr.mgh.harvard.edu/). Cortical data were co-registered to each subject’s native surface via boundary based registration^55^, then further transformed to the fsaverage6 surface via a surface-based registration^56,57^. Surface-based spatial smoothing was then applied at FWHM = 6 mm (twice the voxel resolution). For the movie-watching runs only, volumetric data were also co-registered to each subject’s anatomical T1 image via boundary based registration and then onto the MNI152 standard brain via FSL’s FNIRT tool^58^. Volume-based spatial smoothing was then applied at FWHM = 6 mm. The volumetric data were finally restricted to a subcortical grey-matter mask generated from Freesurfer’s *Aseg* atlas^59^ comprising the following labels: cerebellum grey matter, thalamus, caudate, putamen, pallidum, hippocampus, amygdala, accumbens area, and ventral diencephalon.

#### Visual field mapping

We performed a travelling wave analysis^17^ of the retinotopy data registered to the cortical surface using the *3dRetinoPhase*^60^ command in AFNI^61^ (https://afni.nimh.nih.gov/). This yielded phase maps representing the eccentricity and polar angle tunings of each surface vertex. The polar angle map was used to define individualised V1 regions of interest (ROIs) by tracing along the phase reversals. The eccentricity and polar angle maps restricted to the V1 ROIs also provided a ground-truth estimate of the retinotopic maps against which the connectopic maps could be benchmarked.

### Experiment 2

Our second experiment presents preregistered analyses of a larger dataset comprising both movie-watching and resting-state data obtained from the Human Connectome Project.

#### Dataset

We obtained visual field mapping, resting-state, and movie-watching 7T MRI data from the Human Connectome Project^35^. We used a subset of 176 subjects from the S1200 release who have fully completed all three tasks. Two subjects (126,931 and 745,555) were removed as MSMAll-aligned versions of their datasets were unavailable, leaving a total of 174 subjects; note that this represents a deviation from our preregistration. A list of the subject IDs is provided in Supplementary Table S1. In brief, functional data were acquired on a 7T Siemens Magnetom MRI scanner via a multiband EPI sequence (TR = 1 s, TE = 22.2 ms, voxel resolution = 1.6 mm isotropic). The visual field mapping stimuli comprised a flickering “mashfast” texture of objects superimposed on an amplitude mask background^62^ presented within apertures displaying rotating wedges, expanding/contracting rings, or drifting bars. Resting-state data were acquired in four scan runs each approximately 16 min in duration. The movie-watching stimuli comprised approximately 1 h of short clips, taken from independent films and Hollywood movies^63^, presented across four scan runs. See the WU-Minn HCP S1200 Data Release reference manual for full details.

We performed a series of power analyses using G*Power v3.1^64^ to ensure the sample size would be appropriate. A basic test of our hypotheses is whether the connectopic mapping can accurately predict the retinotopic maps. This can be tested by measuring the prediction accuracies as the absolute correlation between retinotopic and connectopic maps and using a one-sample t-test to compare the values over subjects against zero (see “Methods”—“Statistical Analyses” section below). The smallest effect size obtained from these tests within our exploratory analyses (see “Results”—“Experiment 1”) was Cohen’s *d*_*z*_ = 1.76 (for the prediction of polar angle maps by the RBF kernel PCA). Entering this effect size into G*Power, with a sample size of 174, and an alpha criterion of 0.05, indicated a power of 100% (to within numerical precision) would be achieved. A further critical test is the ability to discriminate prediction accuracies between the different connectopic mapping algorithms. To test this in our exploratory analyses we conducted a two-way repeated-measures ANOVA on the retinotopy correlations with factors for the map and algorithm type. This revealed an effect size of $\eta_{P}^{2}$ = 0.64 (Cohen’s *f* = 1.33) for the main effect of algorithm type. We entered this effect size into G*Power, listing the effect size specification “as in SPSS”, with 10 measurements (factor levels—the number of algorithms), an alpha criterion of 0.05, and a sample size of 174. This again indicated a power of 100% (to within numerical precision) would be achieved.

#### Pre-processing

Visual field mapping, resting-state, and movie-watching datasets all followed the HCP minimal pre-processing pipeline including FIX-denoising. In brief, this includes gradient distortion correction, motion correction, high-pass temporal filtering with a cutoff of FWHM = 2355 s, and ICA denoising via FSL’s MELODIC tool. No slice timing correction was applied due to the relatively fast TR. Cortical data were registered to the HCP’s fsLR32k standard surface^65^, while subcortical data were registered to the MNI brain. Surface registration was performed via a multimodal alignment procedure (MSMAll) which aligns surfaces based on cortical folding plus additional areal features derived from myelin maps, resting-state network maps, and resting-state visuo-topic maps^66,67^. Full details of the HCP pre-processing can be found in^68,69^. We then applied the following further pre-processing steps: timeseries were converted to percentage signal change, and the data were spatially smoothed at FWHM = 3.2 mm (twice the voxel resolution; surface-based for cortical grayordinates and volume-based for subcortical grayordinates). Unlike with the StudyForrest dataset, we did not regress out the mean white-matter and ventricular timeseries as these structures are not included within the grayordinates.

#### Visual field mapping

We fit a population receptive field model to the retinotopy data using the MATLAB analyzePRF toolbox (https://github.com/cvnlab/analyzePRF) and code adapted from^70^. Phase maps were extracted representing the eccentricity and polar angle tunings of each surface vertex, and the polar angle maps were used to define individualised V1 ROIs by tracing along the phase reversals. As before, the eccentricity and polar angle maps also served as ground-truth estimates to benchmark the connectopic maps against.

#### Deviations from preregistration

We note the following deviations from our pre-registered protocol:

- We intended to use all 176 subjects who fully completed the resting-state, movie-watching, and visual field mapping scans. However, we removed two subjects (126,931, 745,555) because MSMAll-aligned versions of their datasets were unavailable, leaving a total of 174 subjects.
- We include additional exploratory analyses of the within-V1 connectopic mapping analyses (Fig. 8; see *Unregistered Analyses*). These explore the effect of including or omitting a PCA transformation of the timeseries prior to calculating the connectivity fingerprints.

### Estimating functional connectivity

Further connectivity analyses followed the same procedures for both experiments. A schematic illustration of the connectivity analysis pipeline is shown in Fig. 1. Movie-watching data (and resting-state data for the HCP dataset) were partitioned into odd and even scan runs to allow cross-validated parameter selection for the dimensionality reduction algorithms (where applicable). Within each data split, the normalised and spatially smoothed timeseries were concatenated over scan runs. Connectivity fingerprints were estimated for each data split following the methods of^22^. The timeseries were split between surface vertices within the V1 ROI versus all surface vertices and subcortical voxels outside the ROI. For the non-V1 timeseries, the number of dimensions (surface vertices plus subcortical voxels) exceeds the number of samples (timepoints). We therefore reduced the dimensionality via a lossless PCA, retaining all available components (one fewer than the number of timepoints) to explain 100% of the variance. This operation amounts to rotating the samples within the feature space and removing the unused dimensions—this aids the computational tractability of later processing stages, but does not incur any loss of information from the non-V1 data. The timeseries were correlated between the V1 vertices and non-V1 principal components, and the correlations were Fisher transformed. This yielded a set of connectivity fingerprints describing the pattern of functional connectivity between each V1 vertex and the rest of the brain. Within this connectivity space V1 vertices are represented as samples and non-V1 principal components as dimensions. The dimensionality of this space was then reduced to extract the connectopic maps (see below).

We also generated further connectivity estimates to test the influence of the source of connectivity signals. In the first two variants, we derived non-V1 timeseries from only the cortical surface vertices or from only the subcortical voxels. In a further two variants, we tested if the functional topography within V1 alone is sufficient to drive the connectopic mapping. Firstly, we repeated the main analyses with non-V1 timeseries derived from surface vertices and subcortical voxels together, but we replaced the timeseries with normally distributed random noise matched in mean and variance to the real timeseries. These noise timeseries will reproduce any global amplitude differences over vertices/voxels but will not contain any consistent temporal variation. Secondly, we conducted a purely within-ROI analysis by correlating timeseries between pairwise combinations of V1 vertices, disregarding the non-V1 timeseries entirely. Note that the lossless PCA compression stage was omitted for this analysis as the number of vertices within V1 is already less than the number of timepoints. Because each V1 vertex is trivially connected to itself (and the resulting perfect correlation would invalidate the Fisher transformation), the diagonal elements of this connectivity matrix were instead set to zero. For all variants, we then repeated our connectopic mapping with the spectral embedding (nearest-neighbour, unweighted), diffusion map, and Isomap algorithms.

### Connectopic mapping

The connectivity fingerprints were reduced in dimensionality to extract the connectopic maps. We always retained the first two components, which are expected to correspond to the eccentricity and polar angle retinotopic maps. Prediction accuracy was assessed by taking the absolute correlation between the retinotopic and connectopic maps—the absolute value was used to account for the sign ambiguity inherent in the connectopic maps. The order of the components is not guaranteed to be consistent over different analyses, so we re-ordered the first two components according to their correlations with the retinotopic maps. Specifically, we calculated the 2 × 2 pairwise correlations between both retinotopic and connectopic maps, then matched the retinotopic/connectopic map pair with the largest absolute correlation, then further matched the remaining map pair. Note that if both connectopic maps are maximally correlated with the same retinotopic map (or vice versa) then the first match will be based on whichever pairing yields the highest correlation—this ensures that only one connectopic map is matched to each retinotopic map.

We employed a number of dimensionality reduction algorithms, including both linear embeddings and non-linear manifold learning techniques. We provide more detail on each of these algorithms in the sections below. Although not an exhaustive list, we have included some of the most commonly used and widely implemented manifold learning algorithms, including those previously used for connectopic mapping such as spectral embedding^22^ and diffusion maps^16^. In the case of spectral embedding, we also considered a number of variants of the algorithm that have been variously employed in the literature^22,24,49^.

All algorithms were implemented using the *scikit-learn* Python module^71^ (https://scikit-learn.org/stable/index.html), except for the diffusion maps algorithm which was implemented using the *pydiffmap* Python module (https://github.com/DiffusionMapsAcademics/pyDiffMap). Where necessary, parameter selection was performed via cross-validation: a Bayesian optimisation algorithm, implemented using the *scikit-optimize* Python module (https://scikit-optimize.github.io/stable/), selected the parameter value(s) that maximised the absolute correlation between retinotopic and connectopic maps (averaged over maps) within one of the data splits. The selected parameters were then applied to connectopic mapping of the other data split. The Bayesian optimisation performed 100 iterations (including 10 random starts) per parameter to be optimised.

#### Spectral embedding

Spectral embedding, also referred to as Laplacian eigenmaps, is a manifold learning technique that finds a lower dimensional embedding preserving local neighbourhood relationships from the original feature space^72^. This technique has previously been used for connectopic mapping in a number of sensory brain regions^22,24,26,28^, including primary visual cortex, and we provide particular focus to it here. First, a neighbourhood graph was constructed denoting which samples fall within the local neighbourhoods of each other sample. This can be performed using a radius approach, in which each neighbourhood is defined by a fixed distance from each sample (denoted by the parameter ε). However, it may be challenging to find an appropriate radius to apply to all neighbourhoods if samples are not evenly distributed throughout the feature space^73^. Alternatively, a nearest-neighbours approach can be used in which each neighbourhood comprises the *k* nearest samples to a given sample—this has the advantage that the neighbourhood extent can vary with the density of the samples. Here, we construct both radius and nearest neighbour graphs based on the squared Euclidean distance between samples in the connectivity space. The neighbourhood parameter (ε or k) was selected via cross-validation. Nearest neighbour graphs were made undirected by considering a pair of samples as being within each other’s neighbourhood if either sample is within the *k* nearest neighbours of the other. This produces *unweighted* graphs, in which each sample is discretely labelled as being inside or outside a given neighbourhood. The graph may also be made *weighted* by setting the non-zero elements of the unweighted graph to some measure of similarity between the samples. Many metrics are available for weighting, though values must be non-negative. Here, we simply use a Pearson’s correlation between samples, rescaled to a zero to one range (i.e., zero represents a perfect negative correlation). We also include a fully-connected version of the weighted graph in which connections are not first restricted to local neighbourhoods and instead all samples are considered connected to all other samples and are only differentiated by the weights^74^. For reference, Haak and colleagues employed weighted radius-neighbourhood graphs^22,28^. Once the neighbourhood graph has been constructed, it is converted to a graph Laplacian, which is then decomposed into its eigenvectors and eigenvalues. Selecting the eigenvectors corresponding to the smallest *m* non-zero eigenvalues yields the connectopic maps (here *m* = 2).

#### Linear embeddings

We employed two linear embeddings: principal and independent components analysis (PCA and ICA). Neither of these algorithms required any parameter selection.

#### Kernel PCA

Kernel PCA provides a non-linear extension of linear PCA using the kernel trick^75^. Many kernels are available, but we employed two of the more commonly used ones. Firstly, we used a second-order polynomial kernel: $k\left( {x,y} \right) = \left( {x^{T} y + 1} \right)^{2}$, where *x* and *y* are a given pair of feature vectors; this kernel had no free parameters. Secondly, we used a radial basis function (RBF) kernel: $k\left( {x,y} \right) = {\text{exp}}\left( { - \gamma \left\| {x - y} \right\|^{2} } \right)$, where γ is a free parameter that determines the spread of the kernel.

#### Locally linear embedding

Locally linear embedding (LLE) derives a series of linear decompositions within local neighbourhoods of the feature space^76^. Provided the manifold is approximately linear within local neighbourhoods, the technique may recover a non-linear embedding over the manifold as a whole. We used a modified variant of the algorithm (MLLE) that is proposed to produce solutions which are more stable and less prone to distortions^77^. The algorithm takes a single free parameter (*k*) determining the neighbourhood size.

#### Local tangent space alignment

Local tangent space alignment (LTSA) is closely related to LLE, and follows the intuition that local hyperplanes drawn at a tangent to the manifold should become aligned relative to one another if the manifold is appropriately unfolded^78^. As with LLE, it requires a free parameter (*k*) determining the neighbourhood size.

#### Isomap

Isomap is a form of multidimensional scaling using a geodesic distance metric^79^. This respects the topology of the manifold and may allow more accurate reconstructions than multidimensional scaling using a standard Euclidean distance metric. First, a nearest neighbour graph was constructed (taking a free parameter *k*) and weighted according to the Euclidean distances between samples. Pairwise geodesic distances between all samples were then calculated from the local Euclidean distances via Dijkstra’s algorithm^80^. Finally, the geodesic distance matrix was submitted to multidimensional scaling.

#### Diffusion maps

Diffusion maps consider the diffusion distances between samples, defined by the transition probabilities between samples on a random walk over the manifold. This algorithm has previously been applied to connectopic mapping both within local regions and over the whole brain^16,27^. First, a nearest neighbour graph is constructed (taking a free parameter *k*). This graph is then weighted by a Gaussian heat kernel: $k\left( {x,y} \right) = {\text{exp}}\left( {\frac{{\left\| {x - y} \right\|^{2} }}{\varepsilon }} \right)$, where *x* and *y* are a given pair of samples, and ε is a free parameter determining the spread of the kernel (not to be confused with the radius parameter of the same name in spectral embedding). From this, a transition matrix can be defined representing the probability of encountering one sample from another on a random walk over the manifold. In this stage, a free parameter (α) can be used to control the influence of the sampling density on the estimation, with values typically set between 0 (maximal influence) and 1 (no influence). Finally, diffusion distances between samples may be derived using the eigenvalues and associated eigenvectors of the transition matrix. Taking the first *m* eigenvectors (here, *m* = 2) yields the embedded space. Euclidean distances in this space approximate the diffusion distances in the original feature space. The diffusion distance reflects the connectivity of samples over the manifold, such that two samples have a small diffusion distance if they are strongly connected. We optimised all three parameters (*k*, ε, and α) via cross-validation.

#### t-SNE

t-distributed stochastic neighbourhood embedding (t-SNE) represents similarities between samples in terms of probabilities, and aims to derive a lower dimensional embedding in which the probabilities are optimally matched to those in the original feature space^81^. Euclidean distances are converted to probability scores via a Gaussian distribution in the original space and a Student t-distribution in the embedded space. t-SNE often provides good reconstructions of the local manifold structure, though may be less sensitive to the global structure. We optimised two parameters: the perplexity (which determines the number of effective nearest neighbours) and the learning rate (which controls the step size of the gradient descent). We initialised the t-SNE with the PCA embedding as this has been suggested to improve reconstruction quality^82^. Because the algorithm is stochastic, a different solution may be obtained each time the algorithm is run; we therefore fit the algorithm five times and selected the one with the smallest Kullback–Leibler divergence.

### Statistical analyses

Prediction accuracy was assessed by taking the absolute correlation between corresponding retinotopic and connectopic maps. As a basic test of performance, we compared the correlations against zero via a series of one-sample t-tests; a Holm-Bonferroni correction^83^ was applied over multiple comparisons within each algorithm (eccentricity and polar angle maps, variants of the algorithm where applicable, and resting-state and movie-watching tasks for Experiment 2).

Further statistical tests employed the same procedures in both experiments, with the exception that tests for Experiment 2 included an additional factor denoting the task (rest, movie-watching). First, we compared correlations within each algorithm. For the spectral embedding algorithm, correlations were entered into repeated-measures ANOVAs with factors for the map type (eccentricity, polar angle), graph type (weighted fully-connected, unweighted radius, weighted radius, unweighted kNN, weighted kNN), and task (Experiment 2 only). For the remaining embeddings, correlations were compared between the map types with paired-samples t-tests in Experiment 1, and with two-way ANOVAs with factors of map type and task in Experiment 2. To compare between algorithms, we entered correlations from each algorithm into further repeated-measures ANOVAs with factors for the map type, algorithm type, and task (Experiment 2 only). For spectral embedding, only the unweighted nearest-neighbour variant was entered into this analysis as an example of a better performing variant.

Finally, the spectral embedding (unweighted kNN), diffusion map, and Isomap algorithms were applied to fingerprints derived from different connectivity sources. To compare between sources, these correlations were entered into repeated-measures ANOVAs with factors for the map type, algorithm type, connectivity source (cortex + subcortex, cortex, subcortex, noise, and within-V1), and task (Experiment 2 only).

In all ANOVAs, effect sizes are reported in units of partial and generalised eta-squared^84,85^. A Greenhouse–Geisser sphericity correction^86^ was applied to all effects. Where necessary, post-hoc analyses were performed via Tukey contrasts^87^. All statistical tests employed an alpha criterion of 0.05 for determining significance.

## Supplementary Information

Supplementary Information.

## Data Availability

All MRI data were obtained from already publicly available repositories: the StudyForrest project (https://www.studyforrest.org/) and Human Connectome Project (https://db.humanconnectome.org/).

## Code availability

All analysis code is available on the OSF (https://osf.io/5vupk/).
