# Supplementary material for: An evaluation of how connectopic mapping reveals visual field maps in V1
Source: Sci Rep. 2022 Sep 28;12:16249. doi: 10.1038/s41598-022-20322-4 (PMC9519585; doi:10.1038/s41598-022-20322-4)
Supplement: Supplementary file 1 — Supplementary Information. [file 41598_2022_20322_MOESM1_ESM.pdf]

An evaluation of how connectopic mapping reveals visual field maps in V1: Supplementary information

Supplementary Table S1. IDs for the 174 subjects obtained from the HCP.

100610	157336	192641	385046	770352
102311	158035	193845	389357	771354
102816	158136	195041	393247	782561
104416	159239	196144	395756	783462
105923	162935	197348	397760	789373
108323	164131	198653	401422	814649
109123	164636	199655	406836	818859
111514	165436	200210	412528	825048
114823	167036	200311	429040	826353
115017	167440	200614	436845	833249
115825	169040	201515	463040	859671
116726	169343	203418	467351	861456
118225	169444	204521	525541	871762
125525	169747	205220	541943	872764
126426	171633	209228	547046	878776
128935	172130	212419	550439	878877
130114	173334	214019	562345	898176
130518	175237	214524	572045	899885
131217	176542	221319	573249	901139
131722	177140	233326	581450	901442
132118	177645	239136	601127	905147
134627	177746	246133	617748	910241
134829	178142	249947	627549	926862
135124	178243	251833	638049	927359
137128	178647	257845	644246	942658
140117	180533	263436	654552	943862
144226	181232	283543	671855	958976
145834	182436	318637	680957	966975
146129	182739	320826	690152	971160
146432	185442	330324	706040	995174
146735	186949	346137	724446	
146937	187345	352738	725751	
148133	191033	360030	732243	
150423	191336	365343	751550	
155938	191841	380036	757764	
156334	192439	381038	765864	

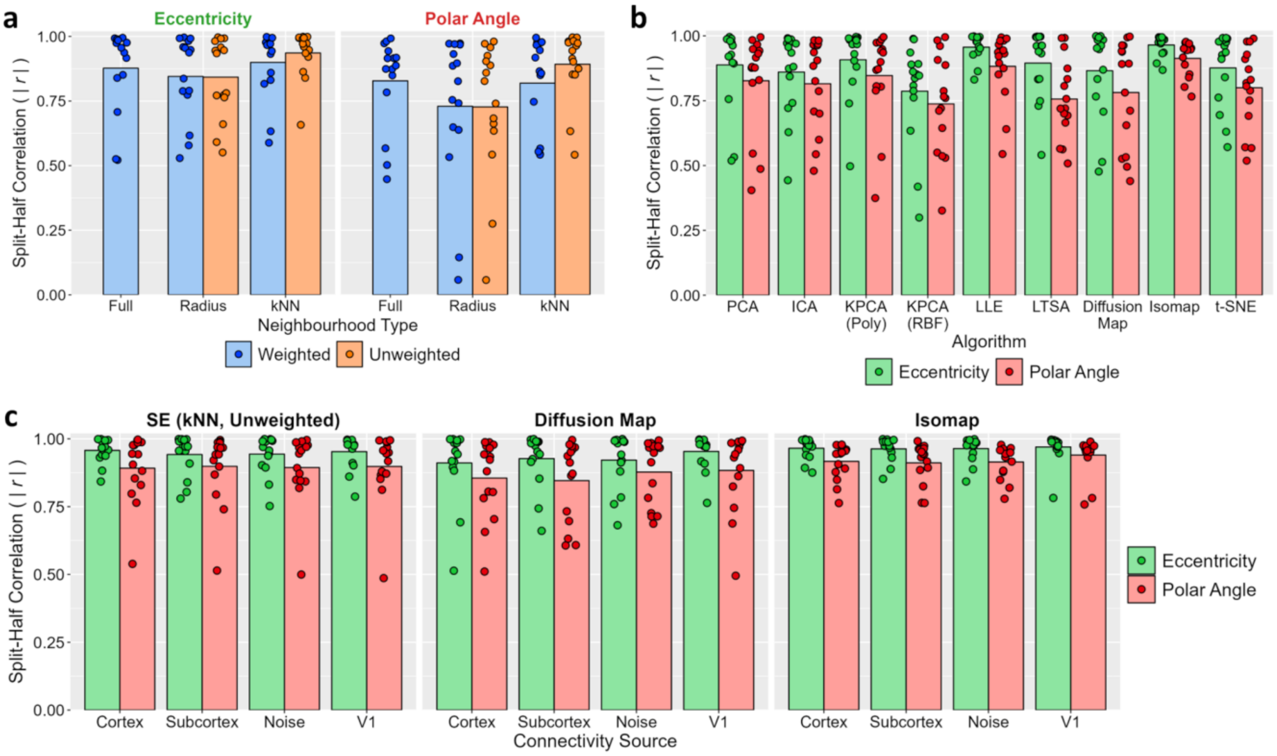

Supplementary Figure S1. Experiment 1: Split-half reliabilities, measured by absolute correlations between connectopic maps across the cross-validation splits. Dot markers indicate per-subject means, and bars indicate group means. (a) All variants of spectral embedding. (b) Other algorithms. (c) Unweighted nearest neighbour variant of spectral embedding, diffusion map, and Isomap algorithms when varying the source of the connectivity fingerprints.

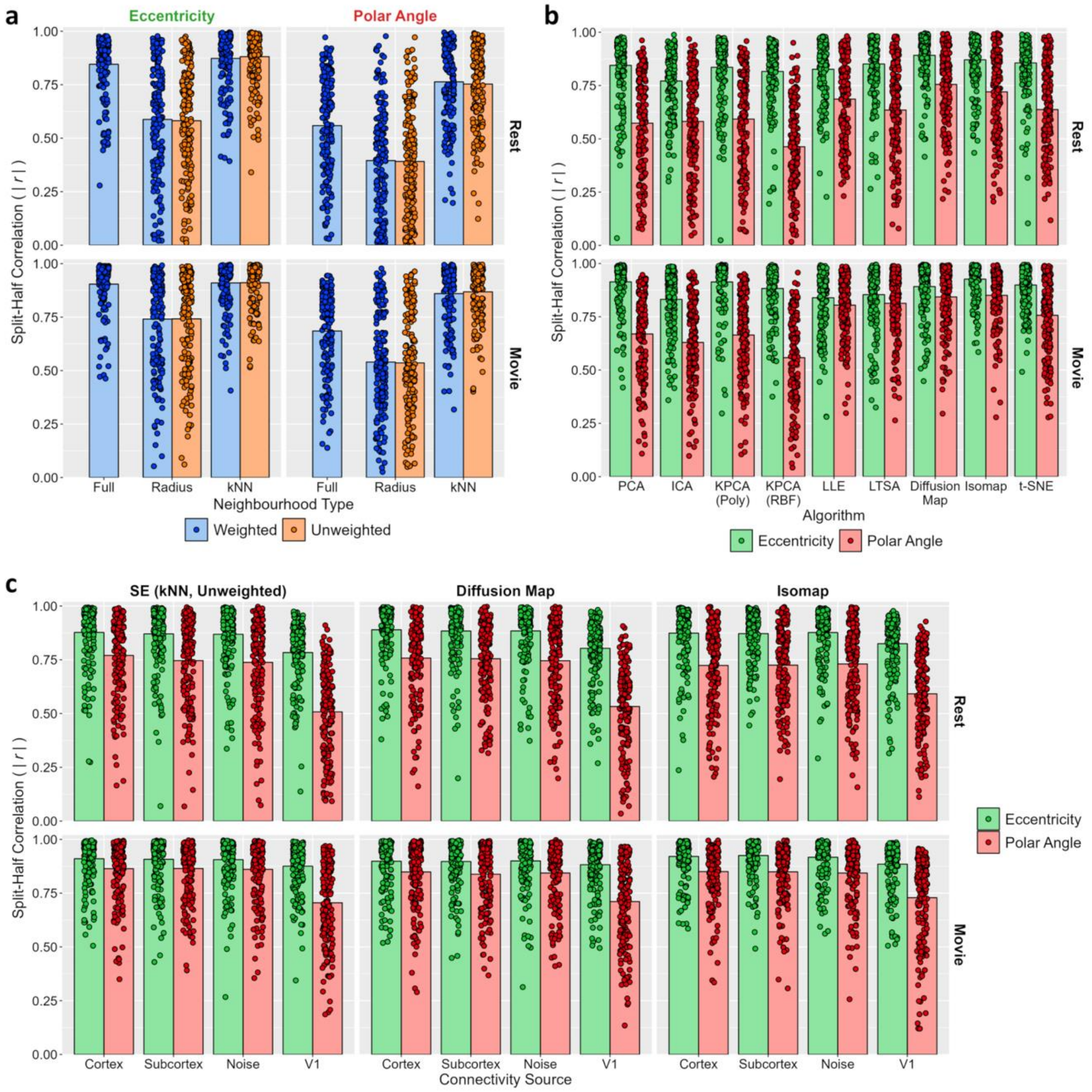

Supplementary Figure S2. Experiment 2: Split-half reliabilities, measured by absolute correlations between connectopic maps across the cross-validation splits. Dot markers indicate per-subject means, and bars indicate group means. Resting-state and movie-watching results are illustrated on top and bottom rows respectively. (a) All variants of spectral embedding. (b) Other algorithms. (c) Unweighted nearest neighbour variant of spectral embedding, diffusion map, and Isomap algorithms when varying the source of the connectivity fingerprints.

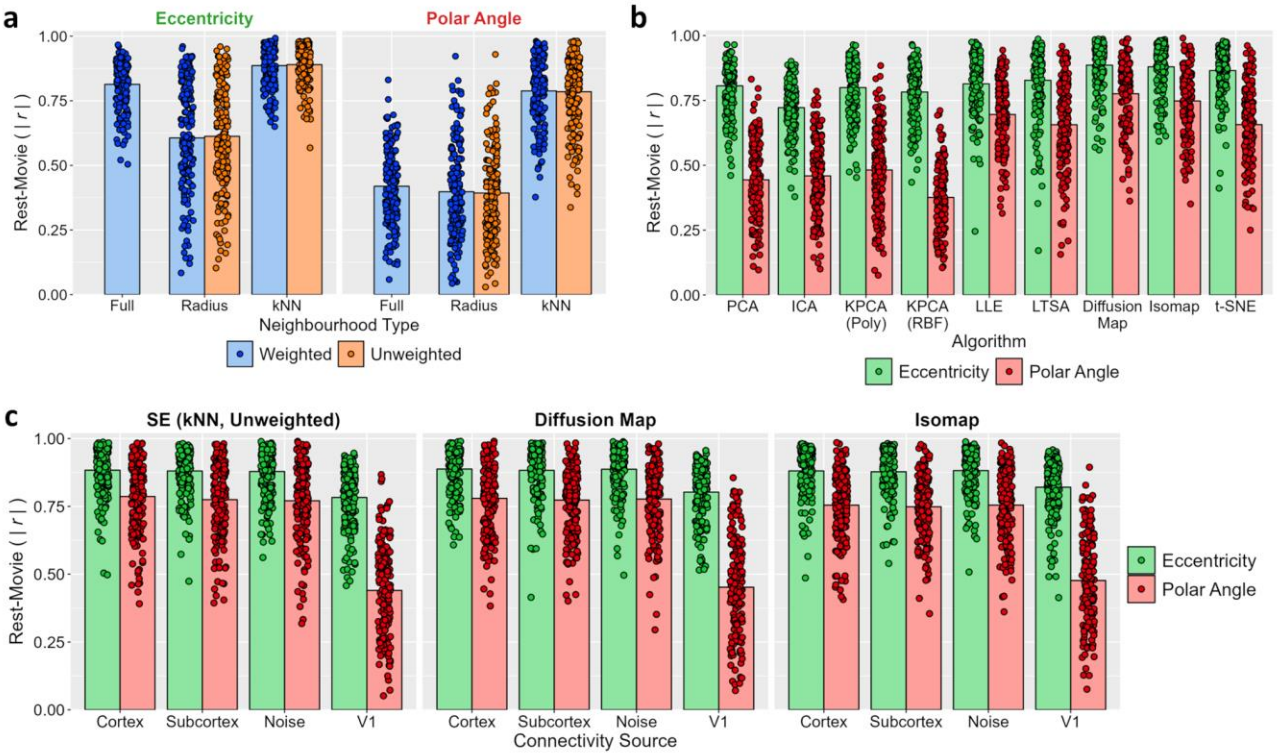

Supplementary Figure S3. Experiment 2: Cross-task reliabilities, measured by absolute correlations between connectopic maps across tasks (rest, movie-watching) and cross-validation splits. Dot markers indicate per-subject means, and bars indicate group means. (a) All variants of spectral embedding. (b) Other algorithms. (c) Unweighted nearest neighbour variant of spectral embedding, diffusion map, and Isomap algorithms when varying the source of the connectivity fingerprints.
